# Supplementary material for: Genome-wide association analysis and replication in 810,625 individuals with varicose veins
Source: Nat Commun. 2022 Jun 2;13:3065. doi: 10.1038/s41467-022-30765-y (PMC9163161; doi:10.1038/s41467-022-30765-y)
Supplement: Supplementary file 1 — Supplementary Information [file 41467_2022_30765_MOESM1_ESM.pdf]

Supplementary Materials

Supplement to:

Waheed-Ul-Rahman Ahmed, Sam Kleeman, Michael Ng, Wei Wang, Adam Auton, 23andMe Research Team, Regent Lee, Ashok Handa, Krina Zondervan, Akira Wiberg, Dominic Furniss. Genome-wide association analysis and replication in 810,625 individuals with varicose veins.

Table of Contents

1. Supplementary Tables

Supplementary Table 1. Fine-mapped SNPs at loci that replicated in 23andMe

Supplementary Table 2. Enriched gene sets from genome-wide gene-based enrichment analysis in MAGMA v1.07

Supplementary Table 3. Odds ratio of varicose veins case/control status in UK Biobank by polygenic risk score decile

Supplementary Table 4. Genetic correlation between varicose veins and other phenotypes

Supplementary Table 5. Results of varicose veins-polygenic score (VV-PRS) analysis

Supplementary Table 6. Summary statistics for bidirectional Mendelian randomization analyses with respect to body mass index, thrombophlebitis, pulmonary embolism, and hernia

Supplementary Table 7. Mendelian diseases associated with varicose veins

Supplementary Table 8. Codes used for varicose veins case definition in UK Biobank

2. Supplementary Figures

Supplementary Figure 1. QQ-plot of observed vs expected P-values in the UK Biobank discovery GWAS

Supplementary Figure 2. Regional Locus Zoom plots of all varicose veins associated loci

Supplementary Figure 3. High concordance between UKB, 23andMe, and FinnGen summary statistics

Supplementary Figure 4. Functional annotation of the 5,315 genome-wide significant SNPs at our 46 replicated loci

Supplementary Figure 5. MAGMA Gene-based association analysis Manhattan plot

Supplementary Figure 6. MAGMA tissue expression analysis

Supplementary Figure 7. Overview of Quality Control (QC)

1. Supplementary Tables

Supplementary Table 1. Fine-mapped SNPs at loci that replicated in 23andMe. 18 probable functional SNPs were discovered from fine-mapping performed across all replicated loci with posterior probability >95%, using Polyfun-SuSiE fine-mapping.

Locus	SNP	CHR	POS	P	Effect	StdErr	A1	A2	Freq	leadSNP	MAF	Posterior probability	SNP_consequence (VEP)
CASZ1	rs11121615	1	10825577	1.40E-158	-0.0148445	0.00055323	C	T	0.69	TRUE	0.340	1.000	promoter
CASZ1	rs6677344	1	10934979	0.01	-0.0014537	0.00056665	A	T	0.29	FALSE	0.317	0.994	intergenic_variant
PROX1	rs17712208	1	214150445	3.00E-06	0.00642	0.00137492	T	A	0.04	FALSE	0.051	0.980	enhancer
PROX1	rs1660373	1	214298120	9.20E-08	0.00278156	0.00052067	A	T	0.55	FALSE	0.417	0.976	enhancer
GATA2	rs7614922	3	128238222	6.80E-19	0.00646877	0.00072868	G	C	0.14	FALSE	0.158	0.959	intergenic_variant
GATA2	rs2713575	3	128294355	7.30E-28	-0.0056096	0.00051267	G	A	0.50	TRUE	0.447	0.990	intron
TBC1D19	rs28558138	4	26818080	2.00E-40	-0.0069013	0.00051846	G	C	0.42	TRUE	0.441	1.000	intergenic_variant
SORBS2	rs11728719	4	186696172	3.00E-11	-0.0039802	0.00059903	A	C	0.24	TRUE	0.208	1.000	non_coding_transcript_variant
CPNE3	rs2304789	8	87567193	2.70E-11	-0.0036015	0.00054045	C	T	0.33	FALSE	0.332	0.986	missense_variant
CPNE3	rs1441249	8	87614243	2.50E-10	0.00322715	0.00050984	T	C	0.53	FALSE	0.482	0.996	TF_binding_site
ETS1	rs79465012	11	128258136	9.70E-11	-0.0063634	0.00098336	C	T	0.07	TRUE	0.052	1.000	intergenic_variant
KLF5	rs41286076	13	73634859	1.10E-11	0.00396095	0.00058332	C	T	0.26	TRUE	0.255	0.992	non_coding_transcript_variant
CPNE2	rs11076178	16	57146402	2.10E-08	0.0044883	0.00080059	C	T	0.11	TRUE	0.126	0.994	promoter
PIEZO1_GALNS	rs112070238	16	88772746	4.50E-07	-0.0025988	0.00051494	C	T	0.44	FALSE	0.411	0.987	5_prime_UTR_variant
PIEZO1_GALNS	rs8053350	16	88794403	5.80E-45	-0.0072434	0.00051481	G	A	0.56	FALSE	0.460	1.000	intron, promoter
NFATC2	rs3787184	20	50157837	3.10E-32	-0.00798	0.00067514	A	G	0.17	TRUE	0.188	0.995	intron, promoter
NFATC2	rs2235856	20	50158473	0.16	0.00073819	0.00052828	C	A	0.64	FALSE	0.344	0.950	intron, promoter
SOX18	rs6062619	20	62683002	1.90E-17	-0.0049799	0.00058603	A	G	0.27	TRUE	0.220	1.000	non_coding_transcript_variant

Supplementary Table 2. Enriched gene sets from genome-wide gene-based enrichment analysis in MAGMA v1.07. The convergence of 15,496 gene sets (15,381from MSigDB v7.0) were tested (See Supplementary Data 3 for all tested gene sets). A Bonferroni-corrected threshold of $P < 3.23\times10^{-6}$ (0.05/15,496) was set, resulting in four significant Gene Ontology (GO) gene sets and two curated gene sets. This analysis was performed using the SNP2GENE tool in FUMA.

**Gene Set**	$\mathbf{N_{genes}}$	**Beta**	$\mathbf{Beta_{STD}}$	**SE**	**P-value**	$\mathbf{P_{bon}}$
GO_bp:go_cardiovascular_system_development	666	0.22331	0.041351	0.039951	1.16×10^{-8}	1.79×10^{-4}
GO_bp:go_tube_morphogenesis	778	0.1964	0.039186	0.037667	9.35×10^{-8}	1.45×10^{-3}
GO_bp:go_blood_vessel_morphogenesis	555	0.21137	0.03584	0.044333	9.39×10^{-7}	1.45×10^{-2}
GO_bp:go_tube_development	956	0.15781	0.03473	0.033944	1.68×10^{-6}	2.60×10^{-2}
Curated_gene_sets:nikolsky_breast_cancer_16q24_amplicon	53	1.7715	0.094095	0.25379	1.53×10^{-12}	2.37×10^{-8}
Curated_gene_sets:cui_tcf21_targets_2_dn	786	0.18957	0.038009	0.03709	1.62×10^{-7}	2.51×10^{-3}

Supplementary Table 3. Odds ratio of varicose veins case/control status in UK Biobank by polygenic risk score decile. The polygenic risk score (PRS) was derived in an independent study sample (FinnGen). OR refers to the odds ratio relative to the first (lowest) PRS decile.

VV-PRS decile	OR	SE	95% CI (lower)	95% CI (upper)
2	1.318	0.043	1.212	1.434
3	1.502	0.042	1.384	1.631
4	1.794	0.041	1.658	1.943
5	1.953	0.040	1.806	2.113
6	2.340	0.039	2.169	2.526
7	2.485	0.039	2.305	2.682
8	2.844	0.038	2.641	3.065
9	3.340	0.037	3.105	3.595
10	4.578	0.036	4.266	4.918

Supplementary Table 4. Genetic correlation between varicose veins and other phenotypes. This analysis was performed using LD score (LDSC) regression, implemented in LD Hub. The traits are shown along with the consortia name, sample size, ethnicity, and PMID of the study from which the LDSC data were derived, the trait category, and the correlation coefficient (r_g). Traits are ranked by P-value, and the twelve traits meeting a Bonferroni-corrected significant threshold of $P < 5.56\times10^{-3}$ are shown. The consortium for each study are as follows: EGG, Early Growth Genetics Consortium; GIANT, Genetic Investigation of ANthropometric Traits Consortium; Alkes, Alkes Group (Harvard T.H Chan School of Public Health); NA, Not Applicable.

Trait 1	Trait 2	Category	r_g	SE	Z-score	P-value	Consortia	Sample Size	Ethnicity	PMID
Varicose veins	Height_2010	Anthropometric	0.16	0.03	5.69	1.28×10^{-8}	GIANT	133,859	European	20881960
Varicose veins	Height; Females at age 10 and males at age 12	Anthropometric	0.21	0.05	4.63	3.59×10^{-6}	EGG	13,960	European	23449627
Varicose veins	Extreme height	Anthropometric	0.17	0.04	4.51	6.36×10^{-6}	GIANT	16,196	European	23563607
Varicose veins	Hip circumference	Anthropometric	0.13	0.03	4.47	7.72×10^{-6}	GIANT	213,038	European	25673412
Varicose veins	Waist circumference	Anthropometric	0.10	0.03	3.80	1.00×10^{-4}	GIANT	232,101	European	25673412
Varicose veins	Child birth weight	Anthropometric	0.21	0.06	3.77	2.00×10^{-4}	EGG	26,836	European	23202124
Varicose veins	Own birth weight	Anthropometric	0.11	0.03	3.71	2.00×10^{-4}	Warrington EGG	321,223	Mixed	31043758
Varicose veins	Offspring birth weight	Anthropometric	0.11	0.03	3.71	2.00×10^{-4}	Warrington EGG	230,069	Mixed	31043758
Varicose veins	Own birth weight	Anthropometric	0.11	0.03	3.64	3.00×10^{-4}	Warrington EGG	286,870	European	31043758
Varicose veins	Birth weight	Anthropometric	0.12	0.04	3.25	1.20×10^{-3}	NA	143,677	European	27680694
Varicose veins	Systemic lupus erythematosus	Autoimmune	0.19	0.06	3.17	1.50×10^{-3}	Alkes Group	23,210	European	26502338
Varicose veins	Body mass index	Anthropometric	0.09	0.03	2.87	4.20×10^{-3}	GIANT	123,912	European	20935630

Supplementary Table 5. Results of varicose vein-polygenic score (VV-PRS) analysis. For phenome-wide VV-PRS analysis, Phecode-level time-to-event data was extracted from UKB phenotype data, and varicose vein-specific phenotypes were removed. For each phenotype, Cox regression was performed for phenotype against Z-scored PRS adjusted for year of birth, sex, genotyping array, recruitment centre and principal components 1-10. Eighteen phenotypes met the phenome-wide significance threshold ($P = 1\times10^{-5}$) in the Cox regression.

**Phenotype**	**Number of events**	**P-value**	**Hazard ratio**
Phlebitis and thrombophlebitis	14,364	4.29E-61	1.15
Unspecified monoarthritis	33,115	7.15E-15	1.04
Obesity	34,193	6.03E-12	1.04
Postphlebitic syndrome	1,452	1.20E-11	1.19
Pulmonary embolism and infarction, acute	8,663	4.72E-11	1.07
Umbilical hernia	6,429	2.59E-08	1.07
Urinary tract infection	40,085	2.97E-08	1.03
Atrial fibrillation and flutter	27,437	3.63E-08	1.03
Prolapse of vaginal walls	15,200	5.66E-08	1.05
Osteoarthrosis NOS	65,054	2.27E-07	1.02
Intestinal malabsorption (non-celiac)	3,774	9.05E-07	0.92
Rheumatism, unspecified and fibrositis	66,165	1.20E-06	1.02
Heart failure NOS	12,311	6.97E-06	1.04
Superficial cellulitis and abscess	24,802	1.75E-05	1.03
Aortic aneurysm	3,471	1.82E-05	1.08
Inguinal hernia	24,196	1.96E-05	1.03
Ventral hernia	5,581	4.33E-05	1.06
Acute sinusitis	19,164	6.34E-05	1.03

Supplementary Table 6. Summary statistics for bidirectional Mendelian randomization analyses with respect to body mass index, thrombophlebitis, pulmonary embolism, and hernia. The primary analysis performed was the inverse-variance weighted (IVW) method, assuming that all instrumental variables are valid. As sensitivity analyses, we additionally performed MR-Egger regression to identify available of significant directional pleiotropy. All analyses were implemented in the TwoSampleMR package for R. Nominally significant associations are highlighted in red (no adjustment for multiple testing).

			IVW			MR-Egger			
**Exposure phenotype**	**Outcome phenotype**	**n_instruments**	**beta**	**se**	**P**	**beta**	**se**	**P**	**pleiotropy_p_value**
Varicose_vein_UKB	Body mass index (ieu-a-2)	72	0.1516	0.1509	0.3153	0.3990735	0.5258	0.4504	0.6245
Body mass index (ieu-a-2)	Varicose_vein_UKB	90	0.0149	0.0031	1.34E-06	0.0156	0.0077	0.0449	0.9126
Varicose_vein_UKB	Thrombophlebitis (finn-a-I9_PHLETHROM)	79	4.2350	1.3724	0.0020	0.7150	4.3663	0.8703	0.3983
Thrombophlebitis (finn-a-I9_PHLETHROM)	Varicose_vein_UKB	2	0.0025	0.0031	0.4270	NA	NA	NA	NA
Varicose_vein_UKB	Venous thromboembolism (finn-a-I9_VTE)	79	1.2247	0.8195	0.1351	0.9585	2.6177	0.7152	0.9150
Venous thromboembolism (finn-a-I9_VTE)	Varicose_vein_UKB	5	0.0023	0.0017	0.1782	0.0010	0.0033	0.7913	0.6547
Varicose_vein_UKB	Hernia (finn-a-K11_HERNIA)	79	0.1024	0.7920	0.8971	-3.7921	2.4850	0.1311	0.1027
Hernia (finn-a-K11_HERNIA)	Varicose_vein_UKB	6	0.0115	0.0056	0.0424	0.0389	0.0243	0.1850	0.3114

Supplementary Table 7. Mendelian diseases associated with varicose veins. This table shows all Mendelian diseases associated with varicose veins, as per the Online Mendelian Inheritance in Man (OMIM) database (https://omim.org, accessed 19th December 2021). For each disease, the PubMed IDs for articles referencing an association with varicose veins is given in the final column.

Disease	Phenotype MIM number	Inheritance	Gene	Locus	PubMed ID
Lymphedema-distichiasis syndrome (LPHDST)	153400	AD	*FOXC2*	16q24.1	11078474; 12114478; 17309653
Cerebral arteriopathy, Autosomal Dominant, with subcortical infarcts and leukoencephalopathy, Type 1 (CADASIL1)	125310	AD	*NOTCH3*	19q13.12	16864835
Lymphatic Malformation 7	617300	AD	*EPHB4*	7q22.1	27400125
Ehlers-Danlos Syndrome, Classic type, 1 (EDSCL1)	130000	AD	*COL5A1*	9q34.3	20635400
Torticollis, keloids, cryptorchidisim, and renal dysplasia (TKCR)	314300	X-linked	unknown	Xq28	4387470; 12725596
Erythrocytosis, Familial, 2 (ECYT2)	263400	AR	*VHL*	3p25.3	14726398
Neutropenia, severe congenital, 4, autosomal recessive (SCN4)	612541	AR	*G6PC3*	17q21.31	20717171
Thauvin-Robinet-Faivre Syndrome (TROFAS)	617107	AR	*FIBP*	11q13.1	26660953
Polymicrogyria with or without vascular-type Ehlers-Danlos Syndrome (PMGEDSV)	618343	AR	*COL3A1*	2q32.2	19455184
Klippel-Trenaunay-Weber Syndrome	149000	unknown	unknown	8q22.3	14327016; 7542989; 8737646
Spastic paraplegia 10, autosomal dominant (SPG10)	604187	AD	*KIF5A*	12q13.1	25352184
Proteus Syndrome	176920	unknown	*AKT1*	14q32.33	11140839
Ehlers-Danlos Syndrome, Spondylodysplastic type, 1 (EDSSPD1)	130070	AR	*B4GALT7*	5q35.3	519906
Megalencephaly-capillary malformation-polymicrogyria syndrome (MCAP)	602501	unknown	*PIK3CA*	3q26.32	18978660
Lymphatic Malformation 6	616843	AR	*PIEZO1*	16q24.3	26333996

Supplementary Table 8. Codes used for varicose veins case definition in UK Biobank. The total number of individuals with each of the diagnostic codes is shown below. A total of 27,165 individuals possessed at least one of the diagnostic codes for varicose veins.

Varicose veins

Source of Data	UK Biobank Data Field	Code	Description	N
Primary ICD-10	41202	I83	Varicose veins of lower extremities	12195
Secondary ICD-10	41204	As above	As above	1168
Primary OPCS	41200	L84	Combined operations on varicose vein of leg	12528
		L85	Ligation of varicose vein of leg	
		L86	Injection into varicose vein of leg	
		L87	Other operations on varicose vein of leg	
		L88	Transluminal operations on varicose vein of leg	
Secondary OPCS	41210	As above	As above	8116
Non-cancer illness (self-report)	20002	1494	Varicose veins	2266
**Operation (self-report)**	20004	1479	Varicose vein surgery	20115
Total (excluding overlaps)				**27165**

2. Supplementary Figures

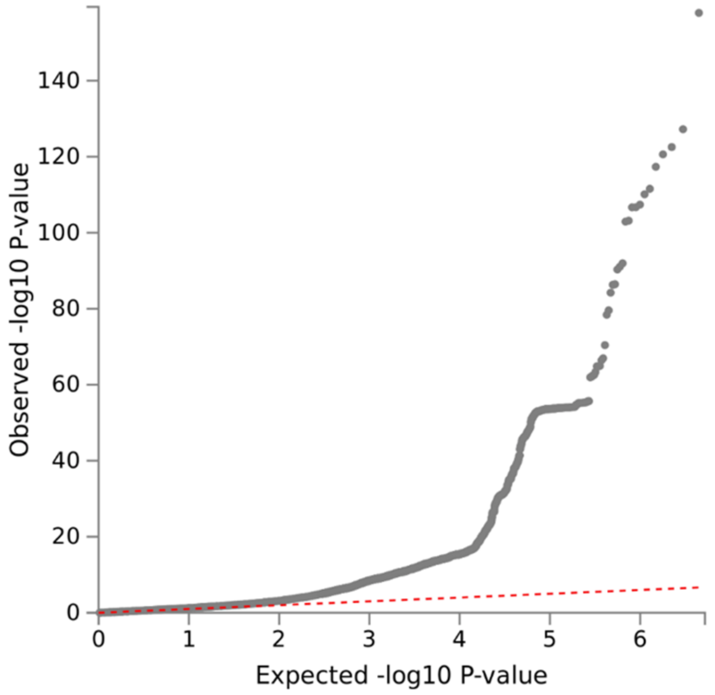

Supplementary Figure 1. QQ-plot of observed vs expected P-values in the UK Biobank discovery GWAS.

Supplementary Figure 2. Regional Locus Zoom plots of all varicose veins associated loci. LocusZoom plots of the 49 independent genome-wide significant SNPs at the 46 replicated varicose veins associated susceptibility loci. Plots are ordered by chromosome number and genomic position. SNP position is shown on the x-axis, and strength of association on the y-axis (-log10 P-value). The linkage disequilibrium (LD) relationship between the lead SNP and the surrounding SNPs is indicated by the r^2 legend. In the lower panel of each sub-figure, genes within 500kb of the index SNP are shown. The position on each chromosome is shown in relation to Human Genome build hg19.

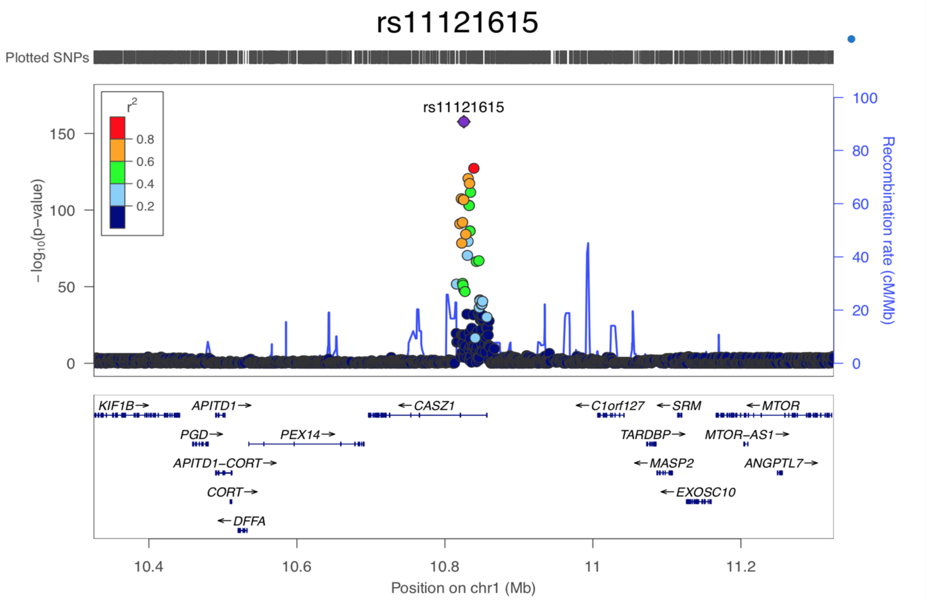

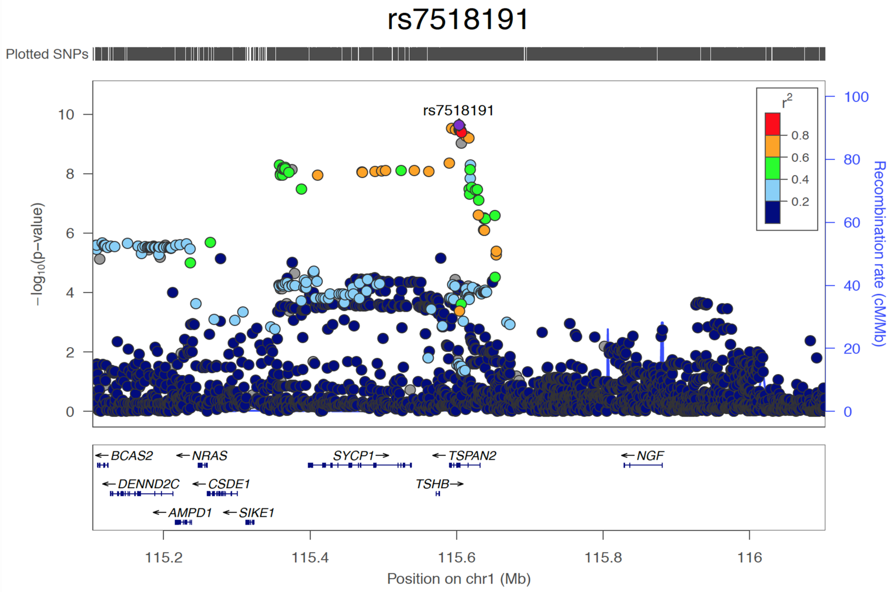

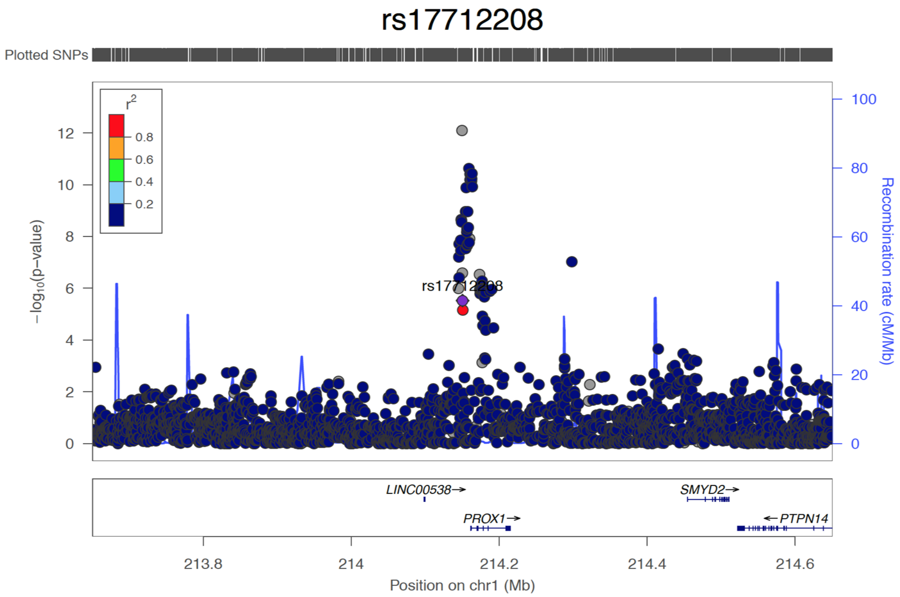
rs17712208
Plotted SNPs
r2
0.8
0.6
0.4
0.2
−log10(p−value)
Recombination rate (cM/Mb)
LINC00538→
SMYD2→
PROX1→
←PTPN14
213.8
214
214.2
214.4
214.6
Position on chr1 (Mb)

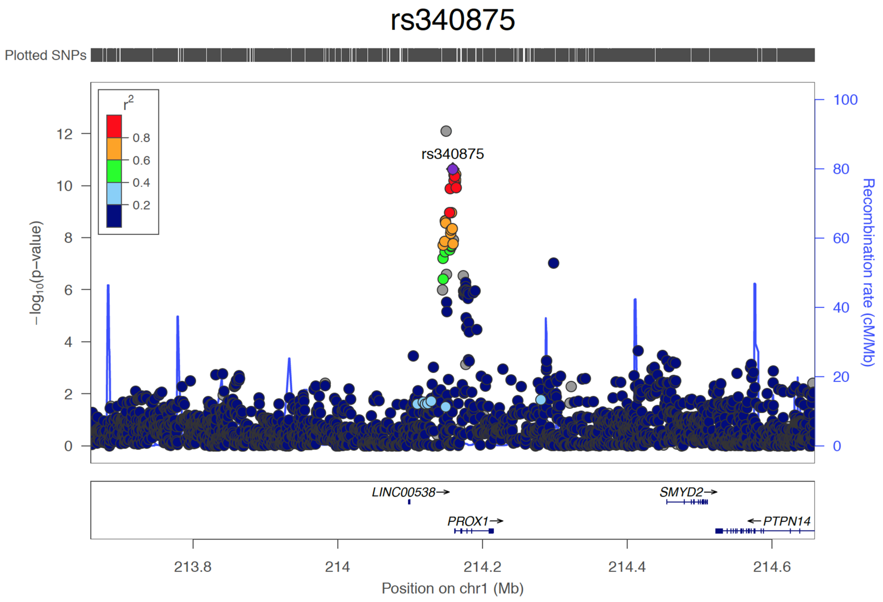
rs340875
Plotted SNPs
r2
0.8
0.6
0.4
0.2
rs340875
−log10(p−value)
Recombination rate (cM/Mb)
LINC00538→
SMYD2→
PROX1→
←PTPN14
213.8
214
214.2
214.4
214.6
Position on chr1 (Mb)

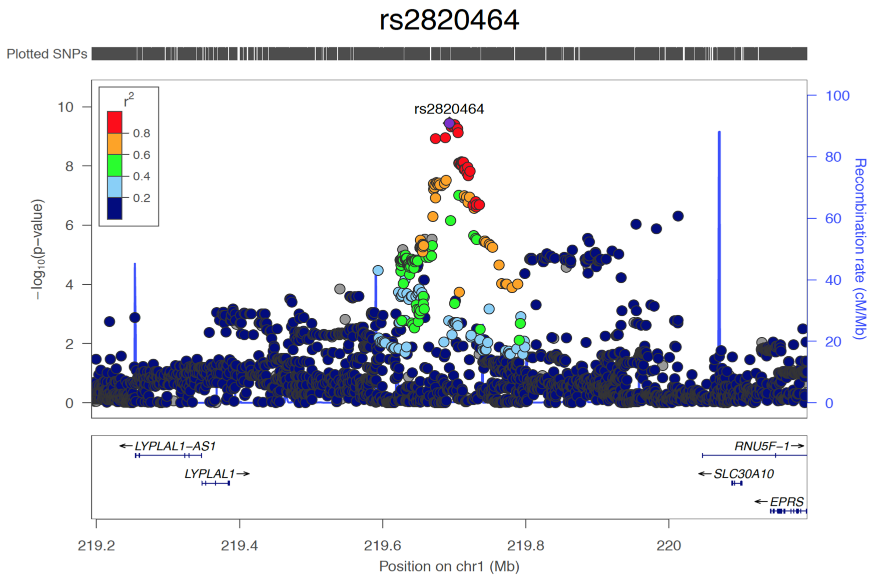
rs2820464
Plotted SNPs
r2
0.8
0.6
0.4
0.2
rs2820464
−log10(p−value)
Recombination rate (cM/Mb)
←LYPLAL1−AS1
RNU5F−1→
LYPLAL1→
←SLC30A10
←EPRS
219.2
219.4
219.6
219.8
220
Position on chr1 (Mb)

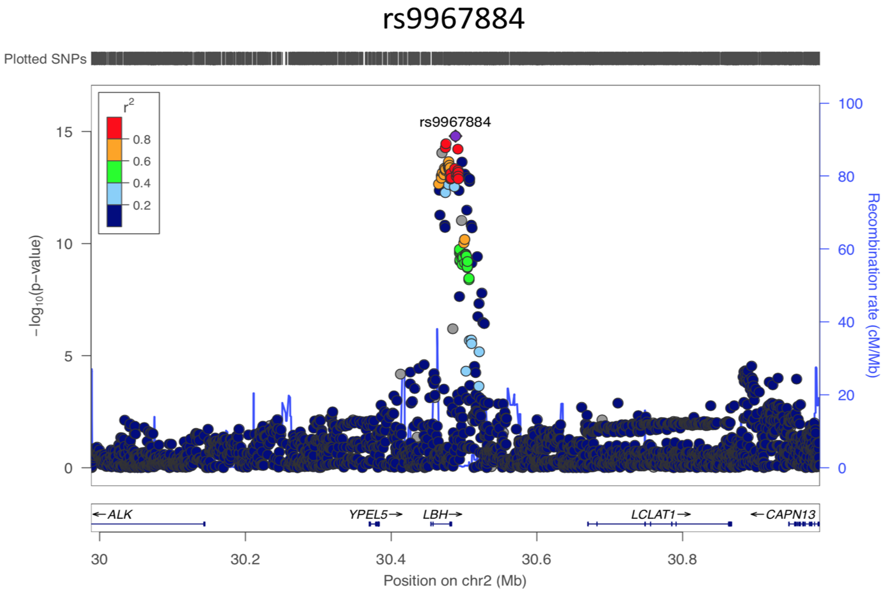
rs9967884
Plotted SNPs
r2
0.8
0.6
0.4
0.2
rs9967884
−log10(p−value)
Recombination rate (cM/Mb)
←ALK
YPEL5→
LBH→
LCLAT1→
←CAPN13
30
30.2
30.4
30.6
30.8
Position on chr2 (Mb)

rs3791679

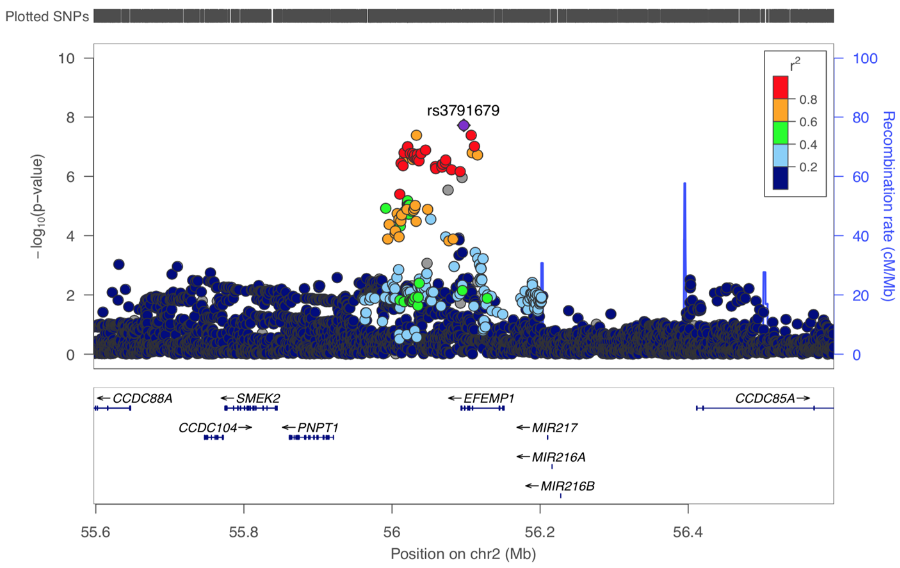

rs2861819

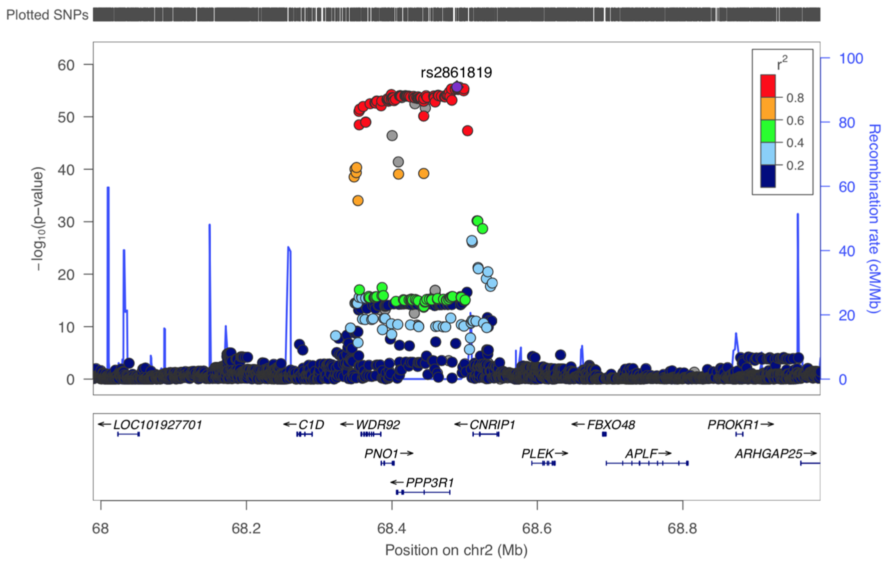

rs4849044

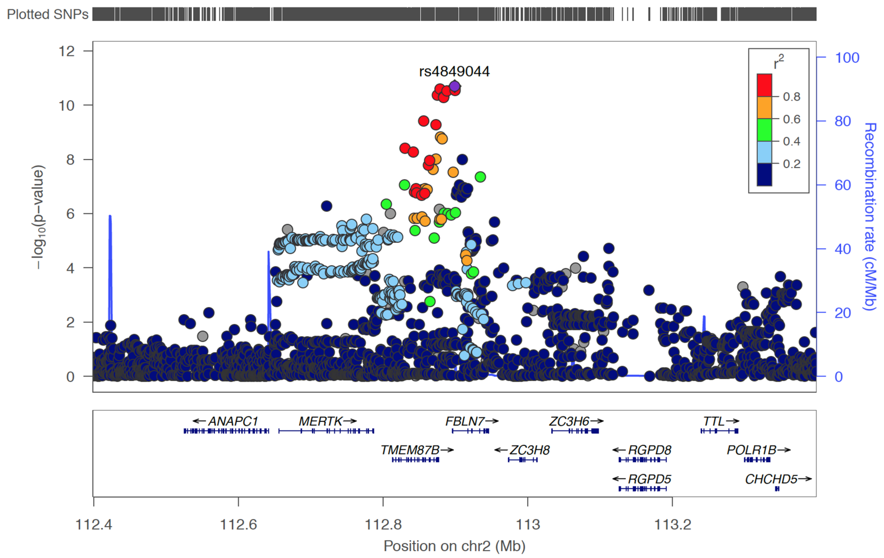

rs17819430

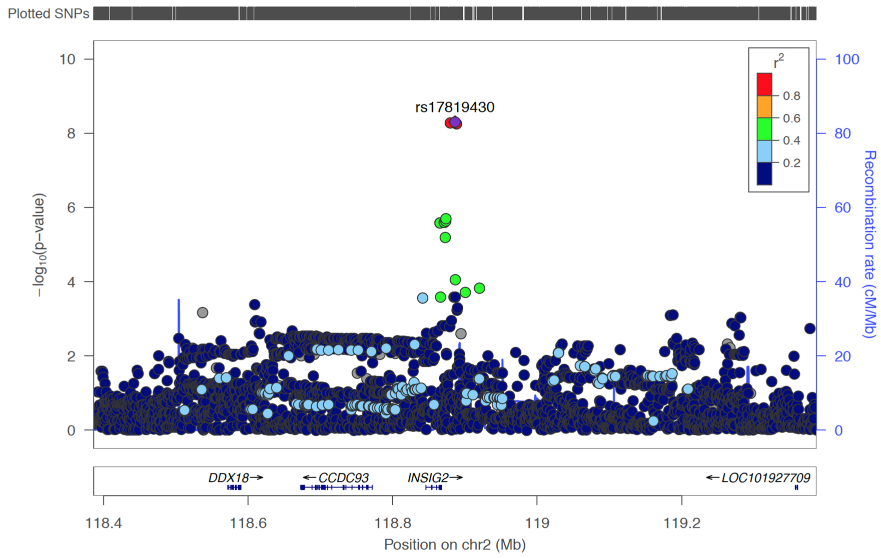

rs55889669

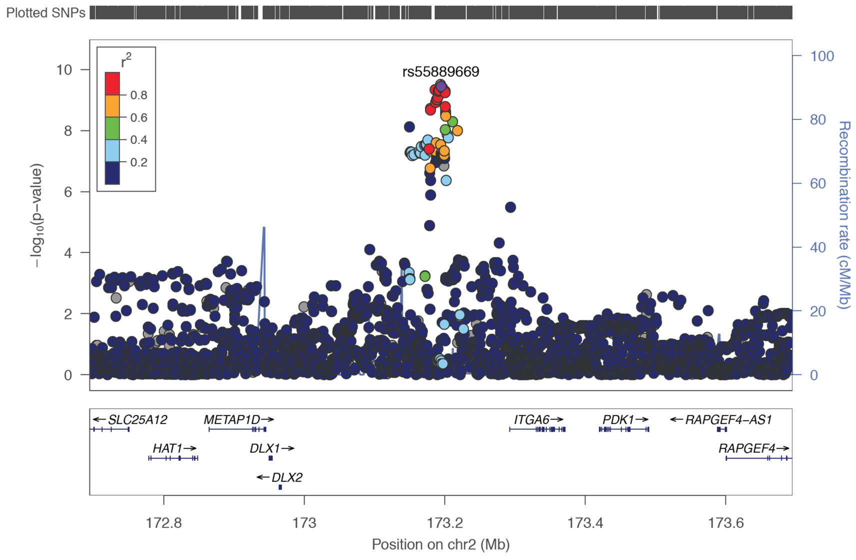

rs844176

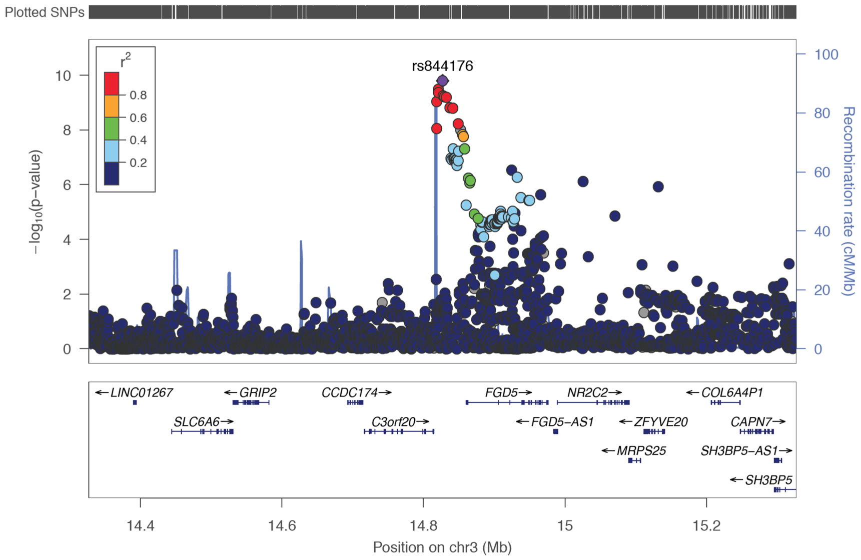

rs2713575

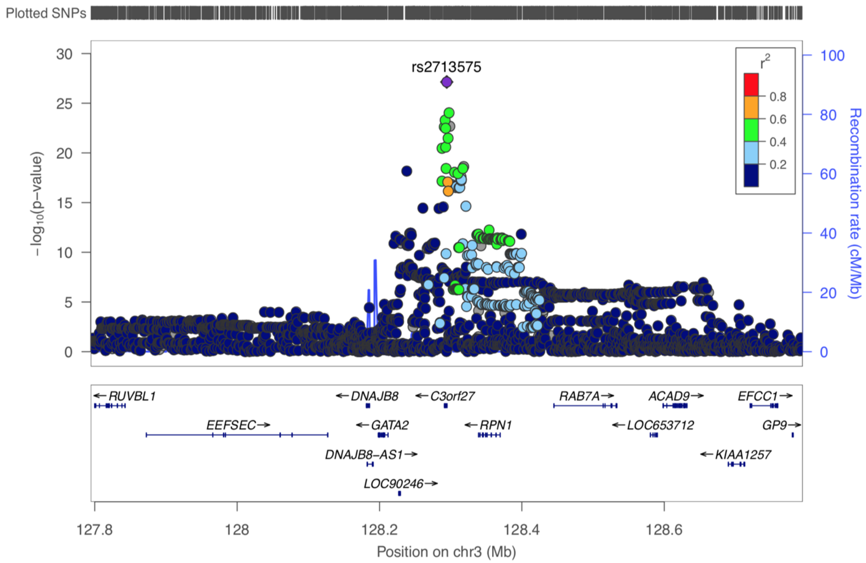

rs9877579

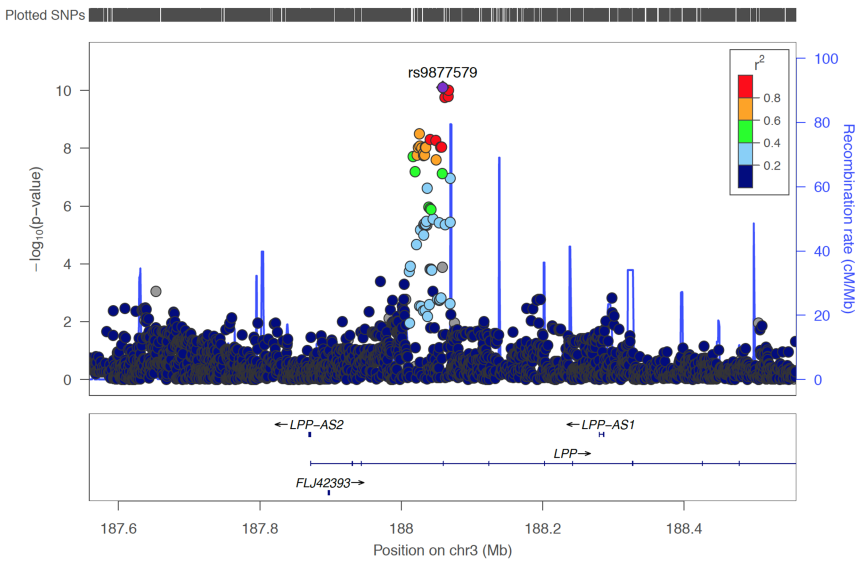

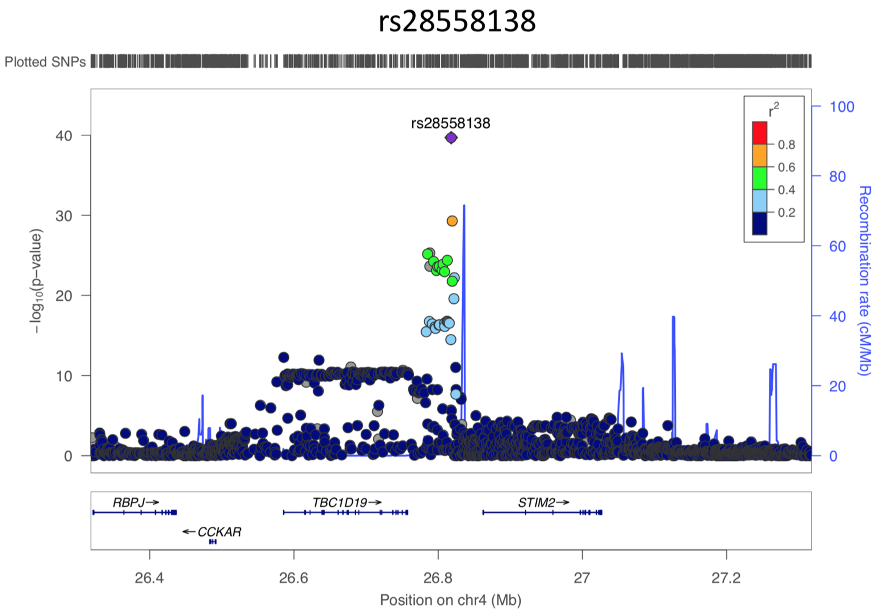
rs28558138
Plotted SNPs
rs28558138
−log10(p−value)
Recombination rate (cM/Mb)
r2
RBPJ
CCKAR
TBC1D19
STIM2
Position on chr4 (Mb)

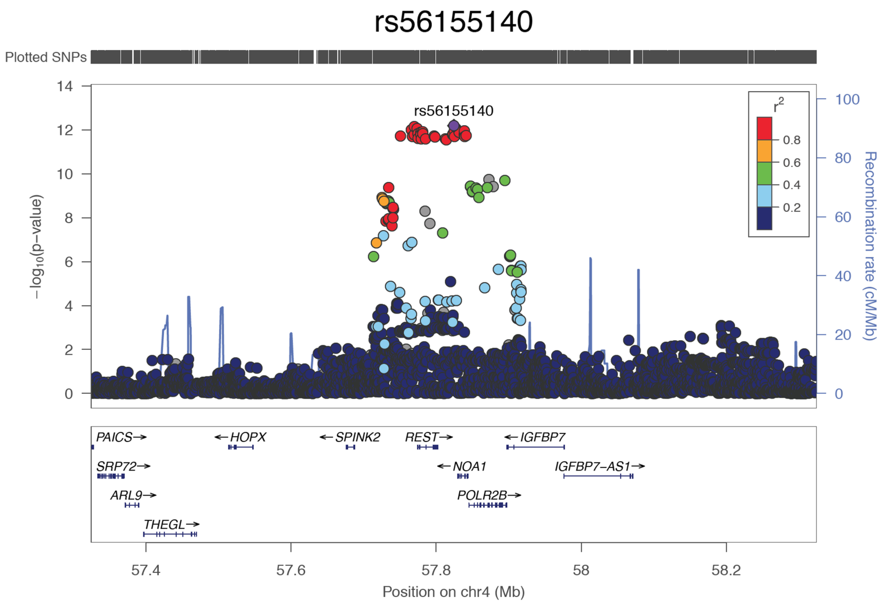
rs56155140
Plotted SNPs
rs56155140
−log10(p−value)
Recombination rate (cM/Mb)
r2
PAICS
SRP72
ARL9
THEGL
HOPX
SPINK2
REST
NOA1
POLR2B
IGFBP7
IGFBP7−AS1
Position on chr4 (Mb)

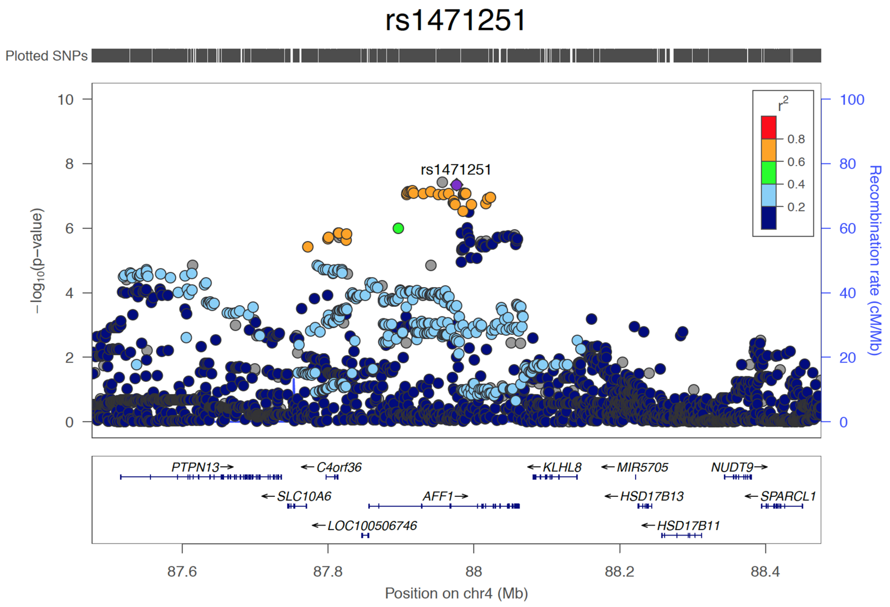
rs1471251
Plotted SNPs
rs1471251
−log10(p−value)
Recombination rate (cM/Mb)
r2
PTPN13
C4orf36
SLC10A6
LOC100506746
AFF1
KLHL8
MIR5705
HSD17B13
HSD17B11
NUDT9
SPARCL1
Position on chr4 (Mb)

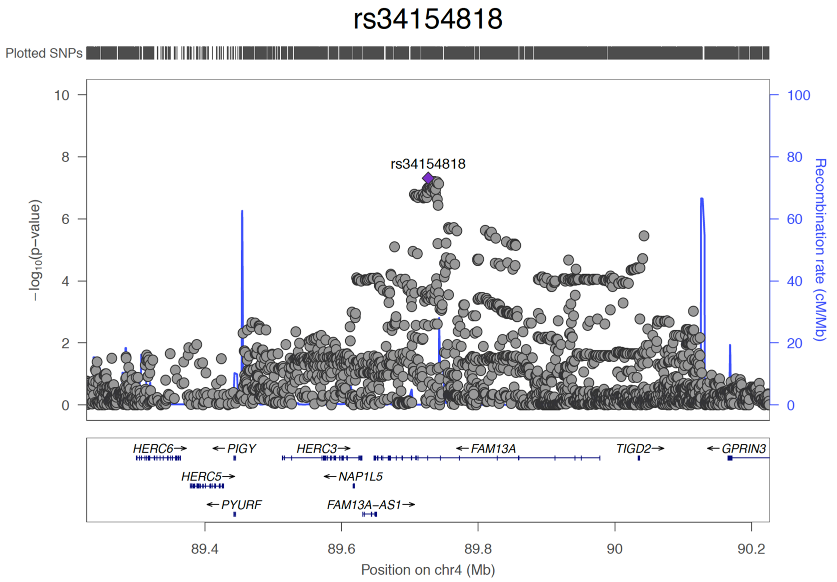
rs34154818
Plotted SNPs
rs34154818
−log10(p−value)
Recombination rate (cM/Mb)
HERC6
HERC5
PYURF
PIGY
HERC3
NAP1L5
FAM13A−AS1
FAM13A
TIGD2
GPRIN3
Position on chr4 (Mb)

rs10007409

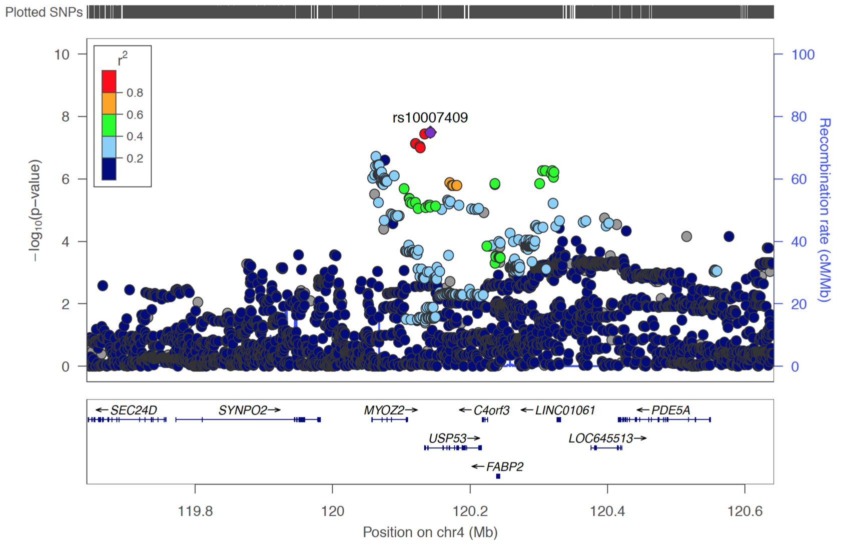

rs11728719

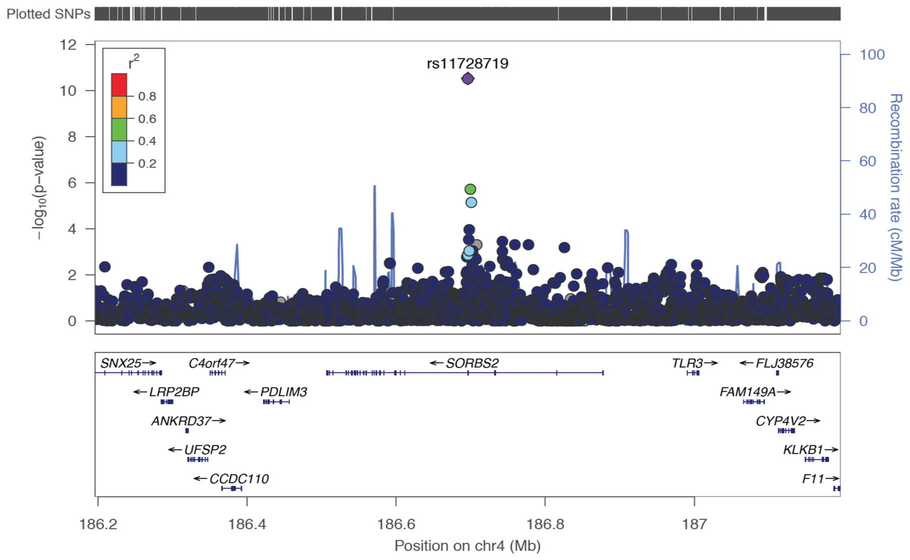

rs57253948

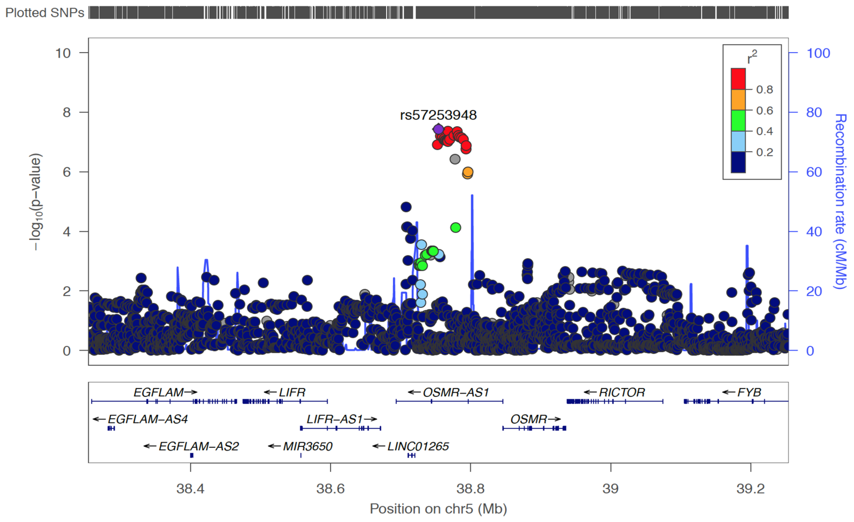

rs3749748

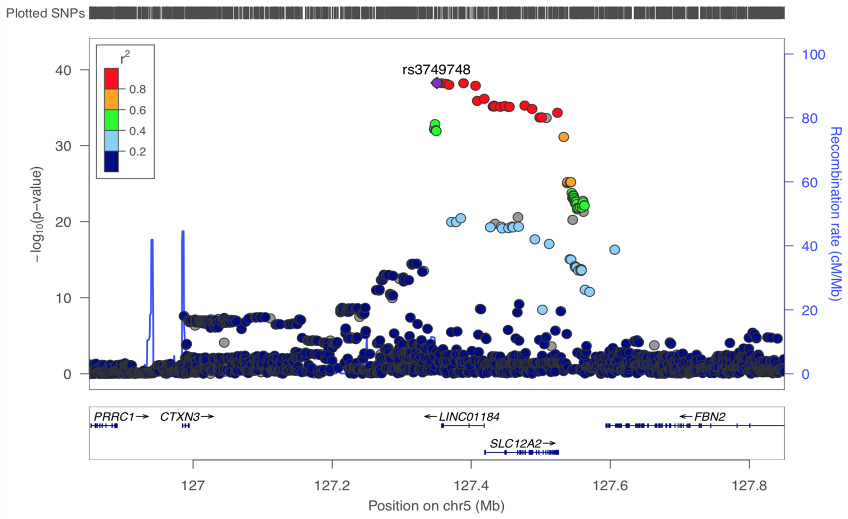

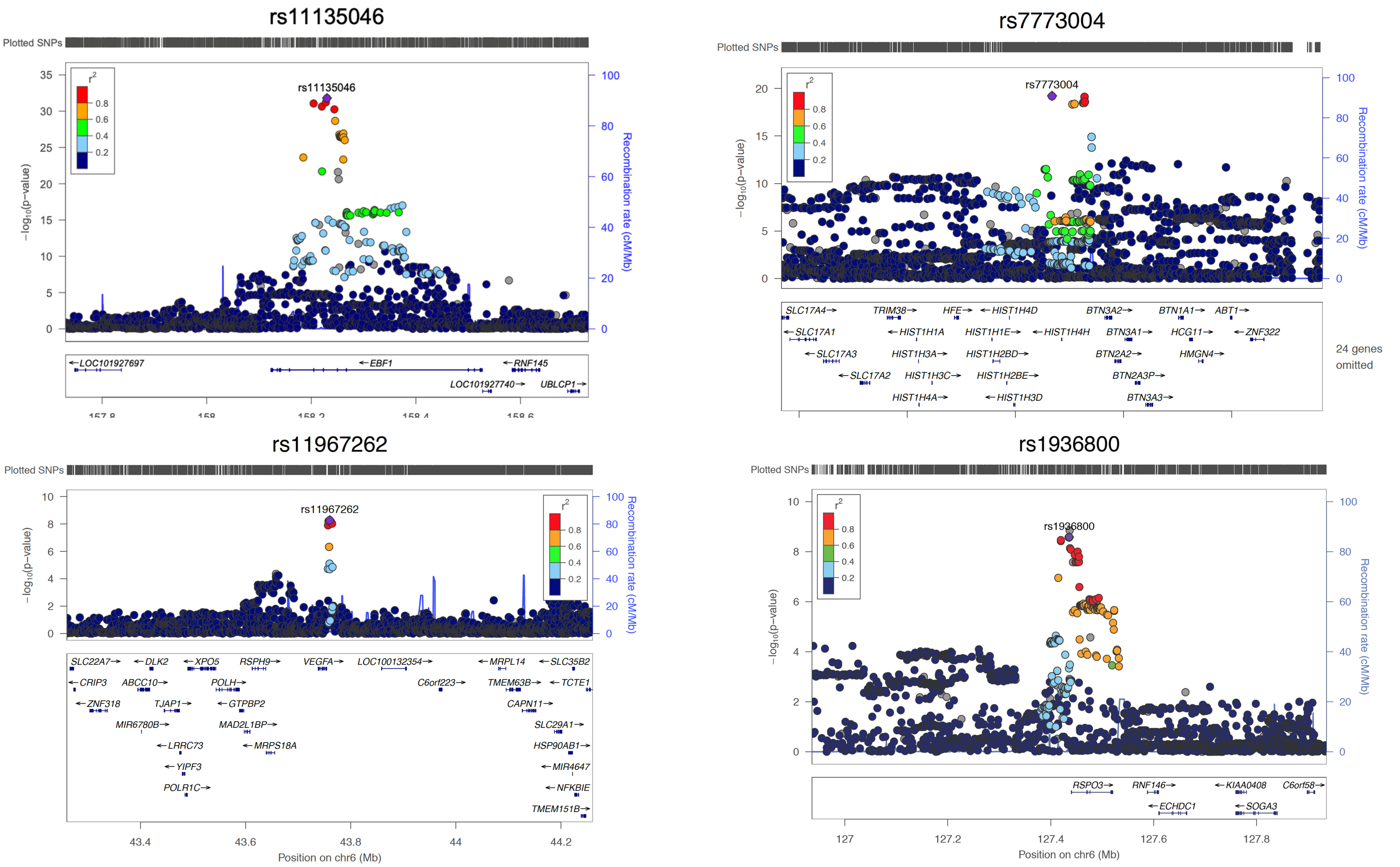

rs11135046
Plotted SNPs
rs11135046
r2
0.8
0.6
0.4
0.2
−log10(p−value)
Recombination rate (cM/Mb)
LOC101927697
EBF1
RNF145
LOC101927740
UBLCP1
rs7773004
Plotted SNPs
rs7773004
SLC17A4
TRIM38
HFE
HIST1H4D
BTN3A2
BTN1A1
ABT1
SLC17A1
HIST1H1A
HIST1H1E
HIST1H4H
BTN3A1
HCG11
ZNF322
SLC17A3
HIST1H3A
HIST1H2BD
BTN2A2
HMGN4
SLC17A2
HIST1H3C
HIST1H2BE
BTN2A3P
HIST1H4A
HIST1H3D
BTN3A3
24 genes omitted
rs11967262
Plotted SNPs
rs11967262
SLC22A7
DLK2
XPO5
RSPH9
VEGFA
LOC100132354
MRPL14
SLC35B2
CRIP3
ABCC10
POLH
C6orf223
TMEM63B
TCTE1
ZNF318
TJAP1
GTPBP2
CAPN11
MIR6780B
MAD2L1BP
SLC29A1
LRRC73
MRPS18A
HSP90AB1
YIPF3
MIR4647
POLR1C
NFKBIE
TMEM151B
Position on chr6 (Mb)
rs1936800
Plotted SNPs
rs1936800
RSPO3
RNF146
KIAA0408
C6orf58
ECHDC1
SOGA3
Position on chr6 (Mb)

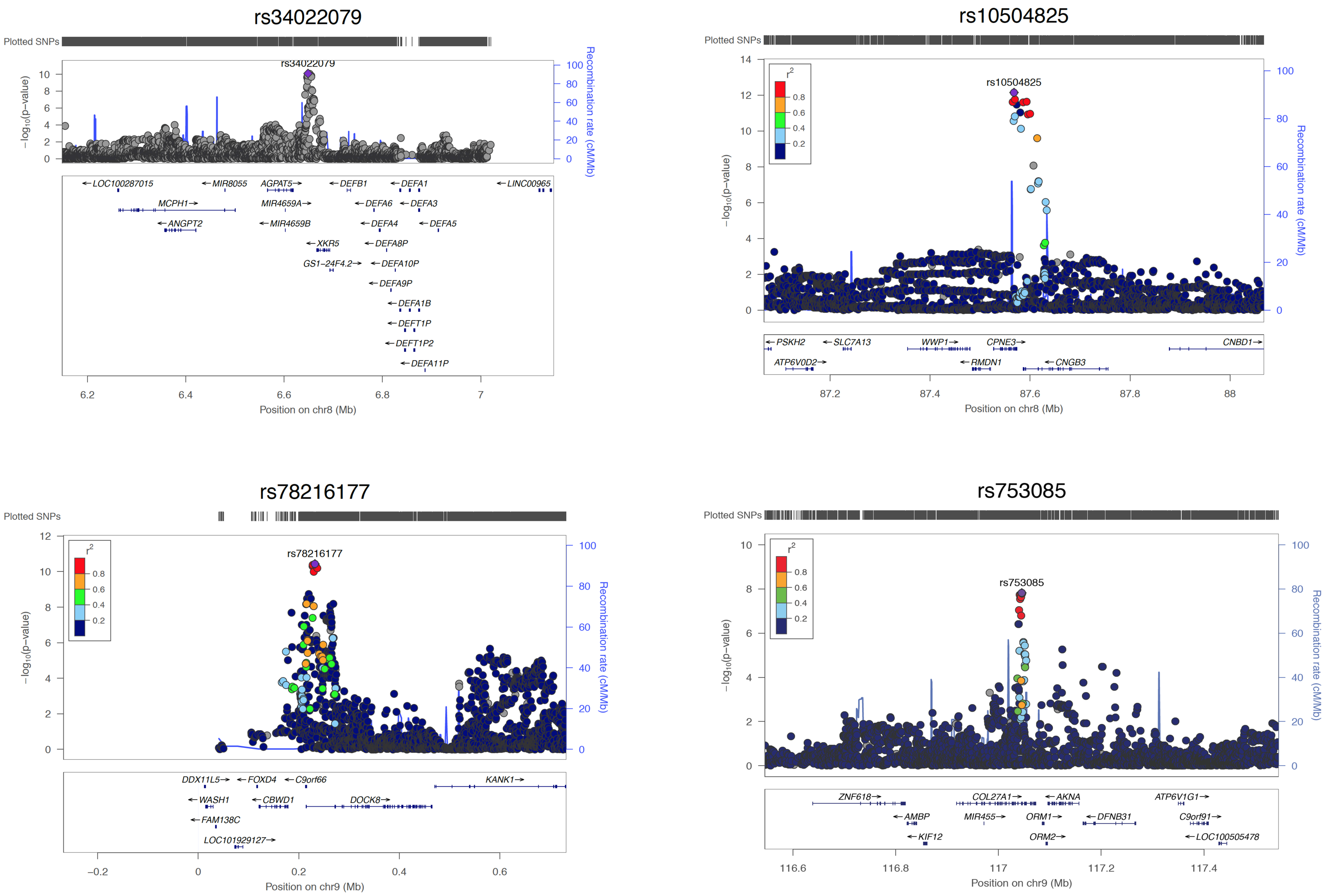

rs34022079
Plotted SNPs
−log10(p−value)
Recombination rate (cM/Mb)
LOC100287015
MIR8055
AGPAT5
DEFB1
DEFA1
LINC00965
MCPH1
MIR4659A
DEFA6
DEFA3
ANGPT2
MIR4659B
DEFA4
DEFA5
XKR5
DEFA8P
GS1−24F4.2
DEFA10P
DEFA9P
DEFA1B
DEFT1P
DEFT1P2
DEFA11P
Position on chr8 (Mb)
rs10504825
r2
PSKH2
SLC7A13
WWP1
CPNE3
CNBD1
ATP6V0D2
RMDN1
CNGB3
rs78216177
DDX11L5
FOXD4
C9orf66
KANK1
WASH1
CBWD1
DOCK8
FAM138C
LOC101929127
Position on chr9 (Mb)
rs753085
ZNF618
COL27A1
AKNA
ATP6V1G1
AMBP
MIR455
ORM1
DFNB31
C9orf91
KIF12
ORM2
LOC100505478

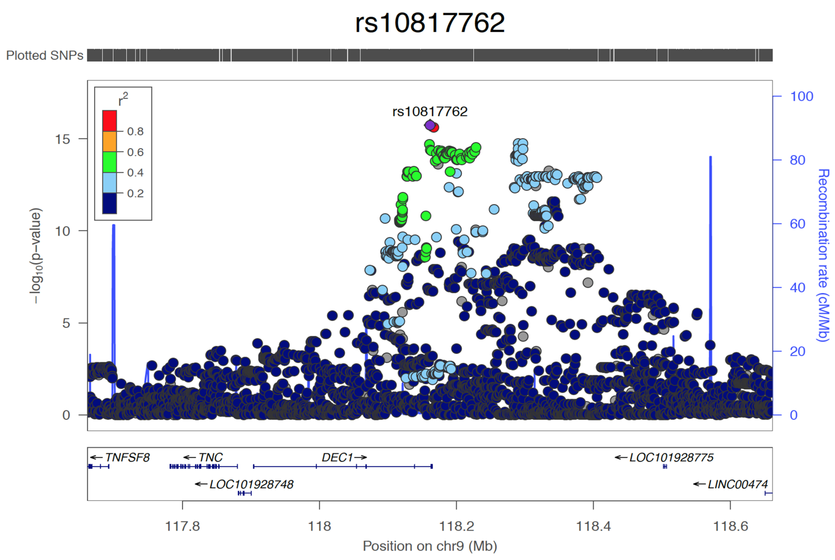
rs10817762
Plotted SNPs
−log10(p−value)
Recombination rate (cM/Mb)
r2
TNFSF8
TNC
DEC1
LOC101928748
LOC101928775
LINC00474
Position on chr9 (Mb)

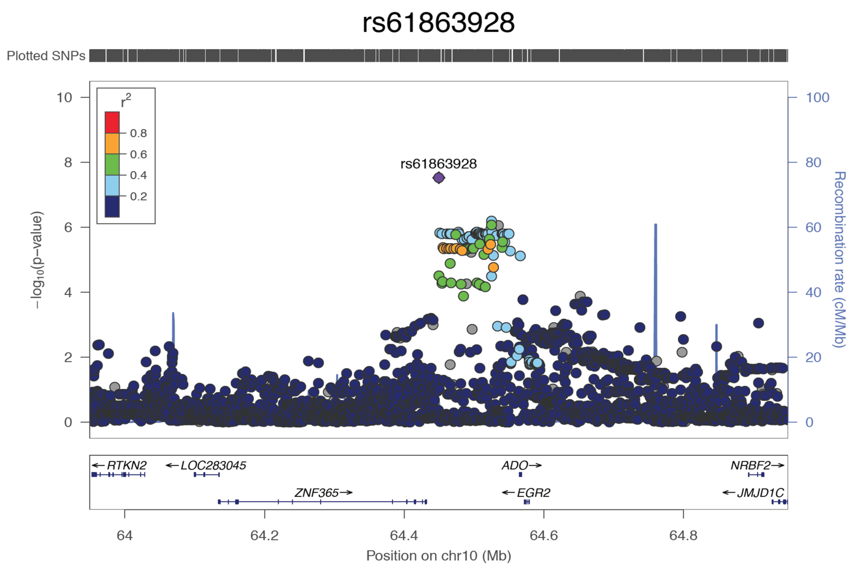
rs61863928
Plotted SNPs
−log10(p−value)
Recombination rate (cM/Mb)
r2
RTKN2
LOC283045
ZNF365
ADO
EGR2
NRBF2
JMJD1C
Position on chr10 (Mb)

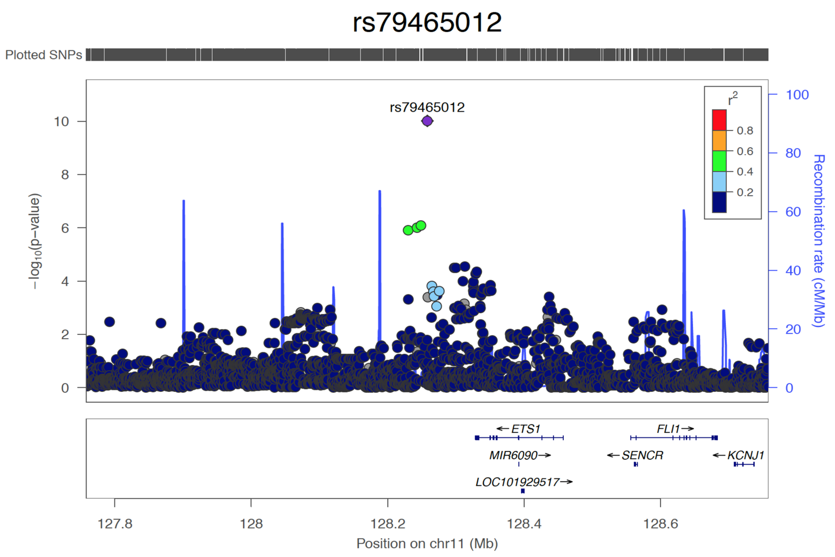
rs79465012
Plotted SNPs
−log10(p−value)
Recombination rate (cM/Mb)
r2
ETS1
MIR6090
LOC101929517
FLI1
SENCR
KCNJ1
Position on chr11 (Mb)

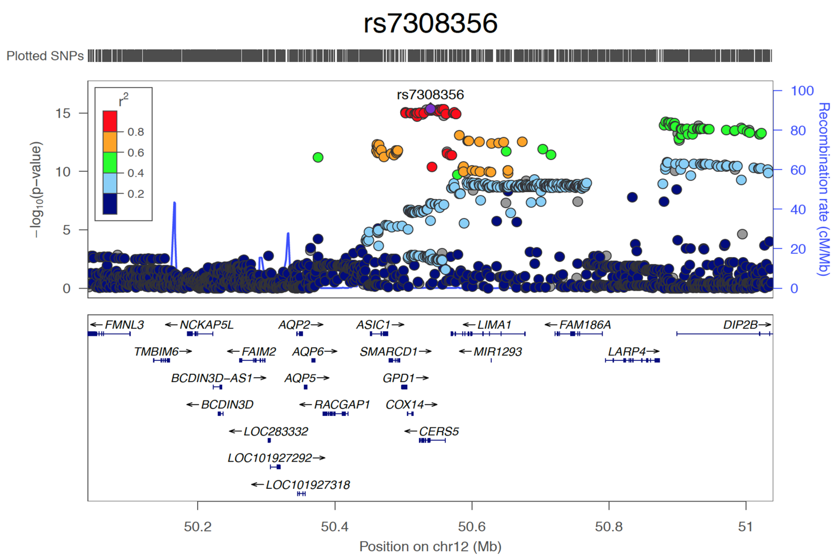
rs7308356
Plotted SNPs
−log10(p−value)
Recombination rate (cM/Mb)
r2
FMNL3
NCKAP5L
AQP2
ASIC1
LIMA1
FAM186A
DIP2B
TMBIM6
FAIM2
AQP6
SMARCD1
MIR1293
LARP4
BCDIN3D−AS1
AQP5
GPD1
BCDIN3D
RACGAP1
COX14
LOC283332
CERS5
LOC101927292
LOC101927318
Position on chr12 (Mb)

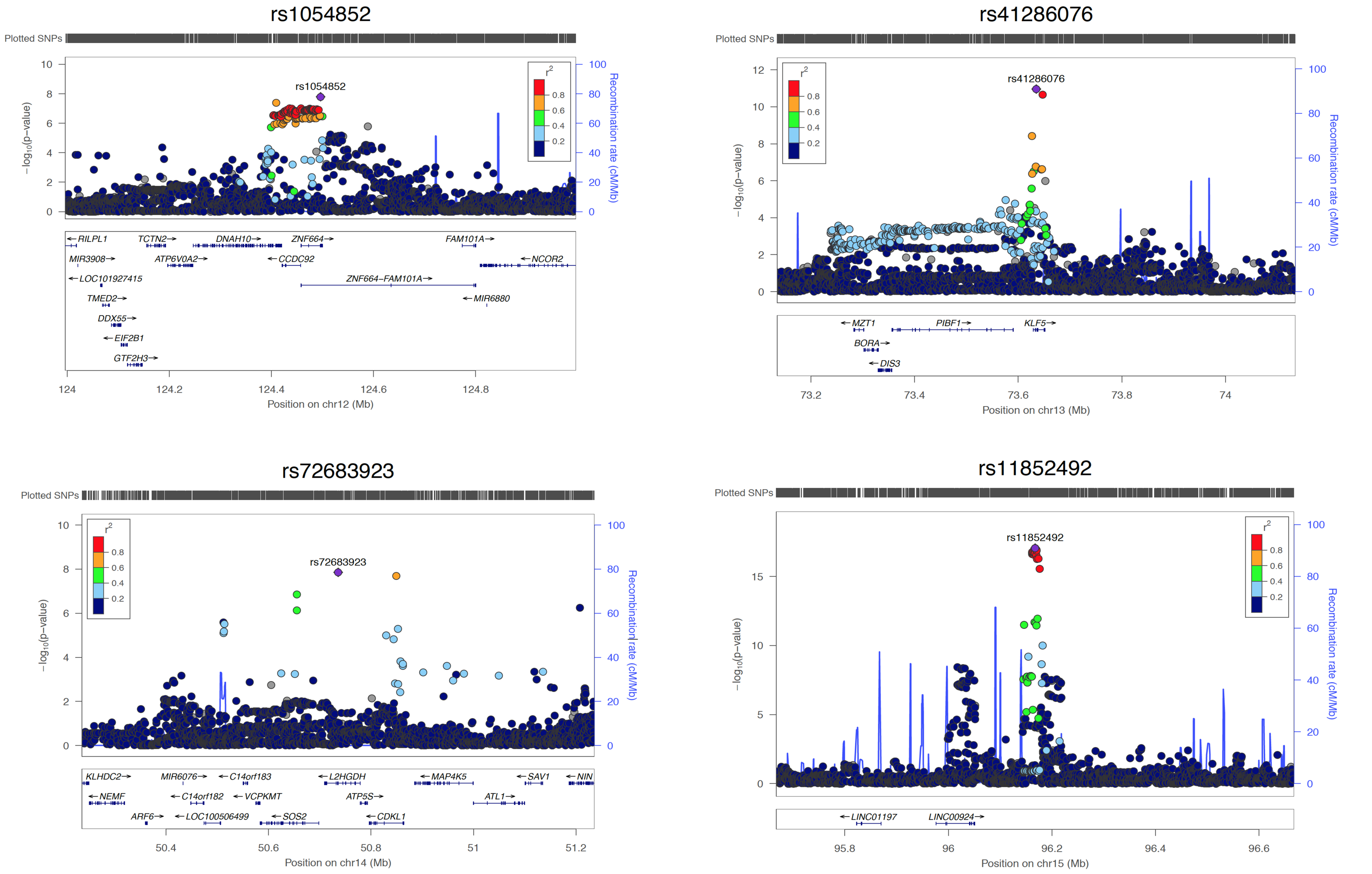
rs1054852
Plotted SNPs
−log10(p−value)
Recombination rate (cM/Mb)
r2
RILPL1
TCTN2
DNAH10
ZNF664
FAM101A
MIR3908
ATP6V0A2
CCDC92
NCOR2
LOC101927415
ZNF664−FAM101A
TMED2
MIR6880
DDX55
EIF2B1
GTF2H3
Position on chr12 (Mb)
rs41286076
MZT1
PIBF1
KLF5
BORA
DIS3
Position on chr13 (Mb)
rs72683923
KLHDC2
MIR6076
C14orf183
L2HGDH
MAP4K5
SAV1
NIN
NEMF
C14orf182
VCPKMT
ATP5S
ATL1
ARF6
LOC100506499
SOS2
CDKL1
Position on chr14 (Mb)
rs11852492
LINC01197
LINC00924
Position on chr15 (Mb)

rs11076178

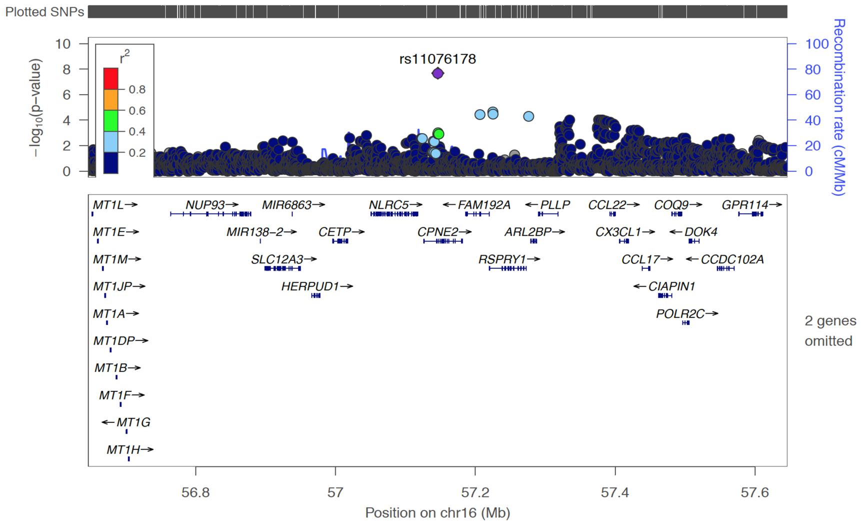

rs111350029

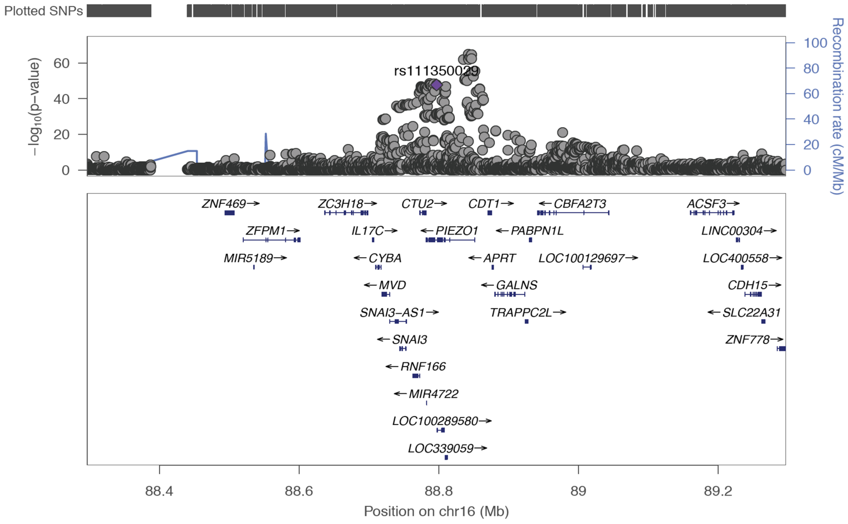

rs11646394

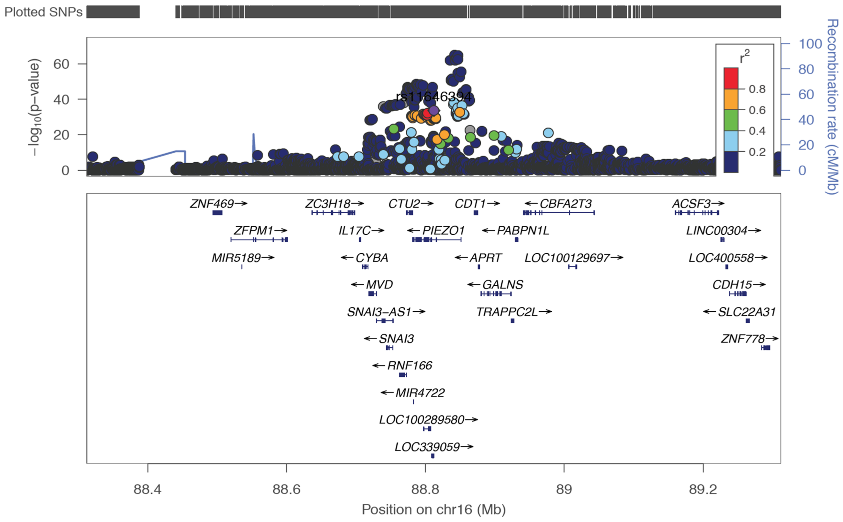

rs2002833

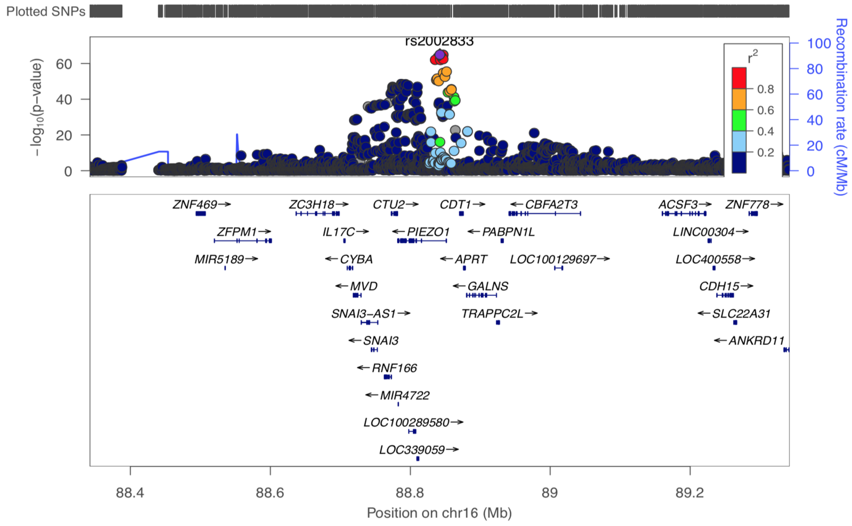

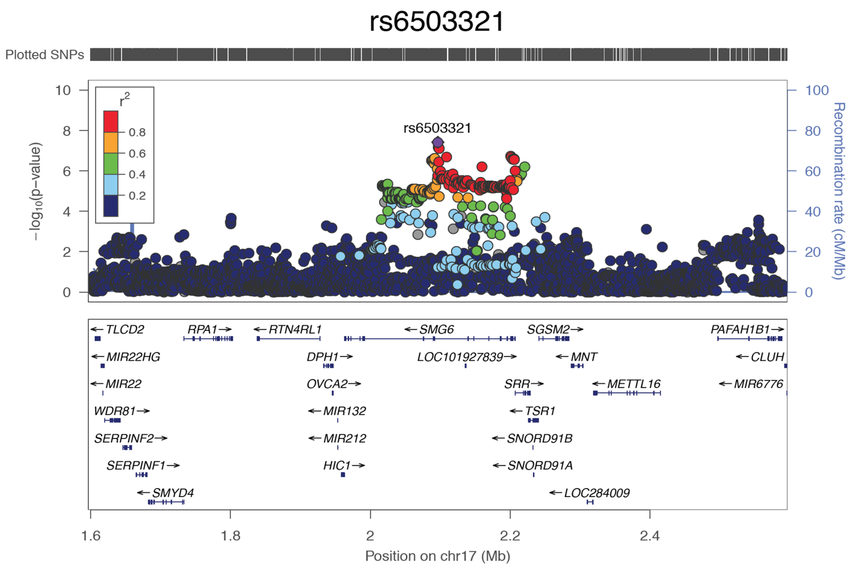
rs6503321
Plotted SNPs
−log10(p−value)
Recombination rate (cM/Mb)
r2
rs6503321
TLCD2
RPA1
RTN4RL1
SMG6
SGSM2
PAFAH1B1
MIR22HG
DPH1
LOC101927839
MNT
CLUH
MIR22
OVCA2
SRR
METTL16
MIR6776
WDR81
MIR132
TSR1
SERPINF2
MIR212
SNORD91B
SERPINF1
HIC1
SNORD91A
SMYD4
LOC284009
Position on chr17 (Mb)

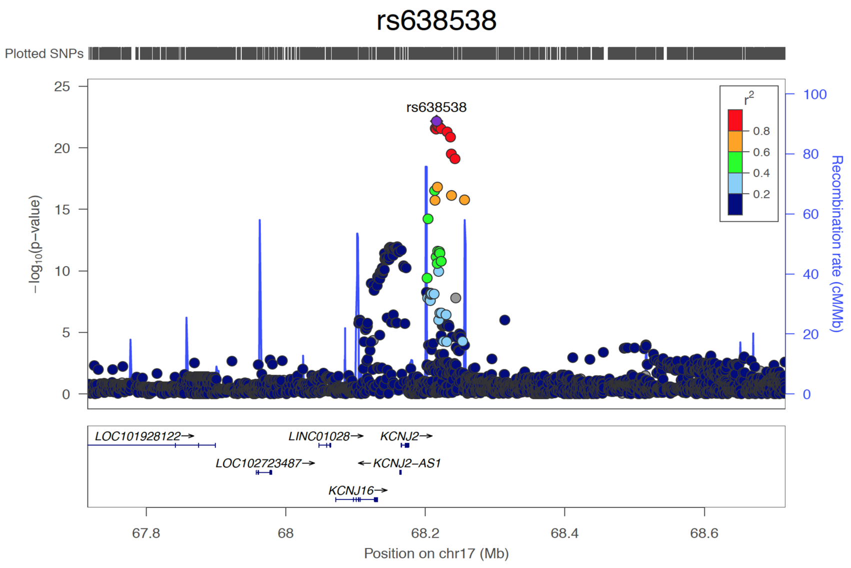
rs638538
Plotted SNPs
−log10(p−value)
Recombination rate (cM/Mb)
r2
rs638538
LOC101928122
LINC01028
KCNJ2
LOC102723487
KCNJ2−AS1
KCNJ16
Position on chr17 (Mb)

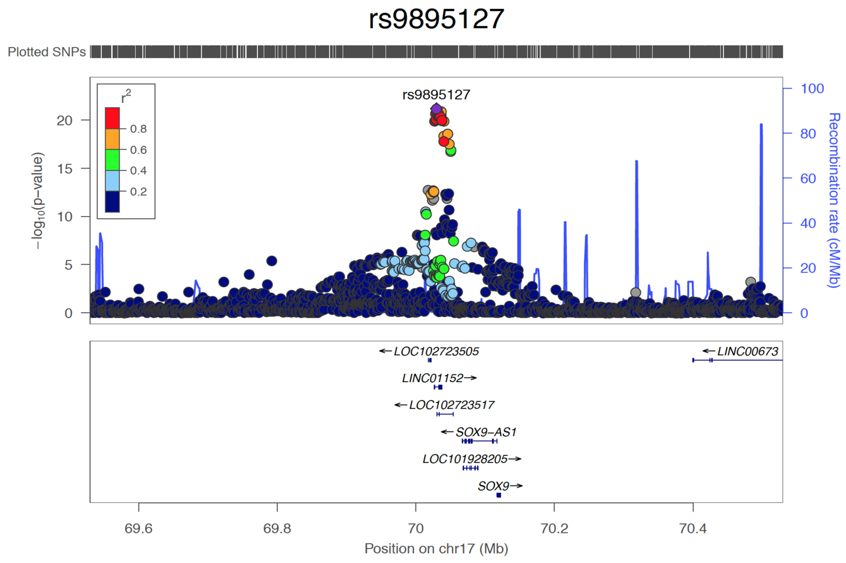
rs9895127
Plotted SNPs
−log10(p−value)
Recombination rate (cM/Mb)
r2
rs9895127
LOC102723505
LINC00673
LINC01152
LOC102723517
SOX9−AS1
LOC101928205
SOX9
Position on chr17 (Mb)

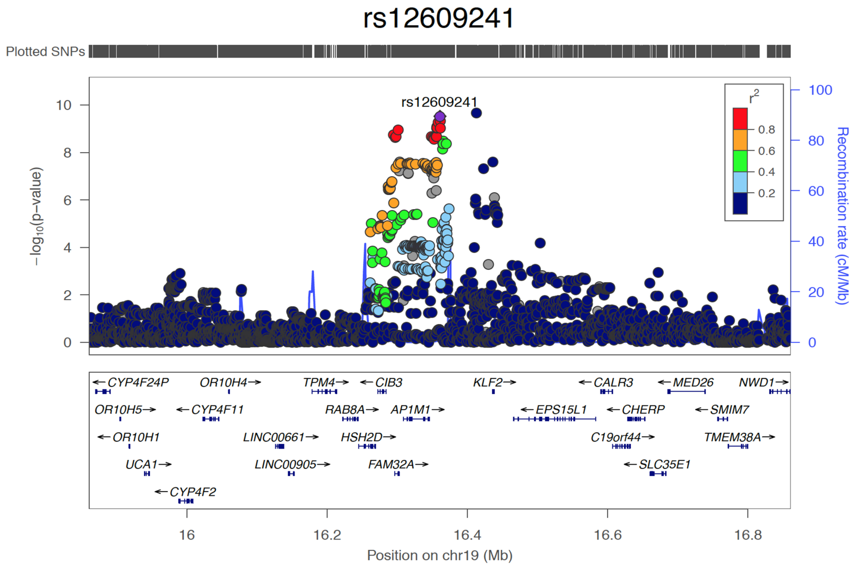
rs12609241
Plotted SNPs
−log10(p−value)
Recombination rate (cM/Mb)
r2
rs12609241
CYP4F24P
OR10H4
TPM4
CIB3
KLF2
CALR3
MED26
NWD1
OR10H5
CYP4F11
RAB8A
AP1M1
EPS15L1
CHERP
SMIM7
OR10H1
LINC00661
HSH2D
C19orf44
TMEM38A
UCA1
LINC00905
FAM32A
SLC35E1
CYP4F2
Position on chr19 (Mb)

rs3787184

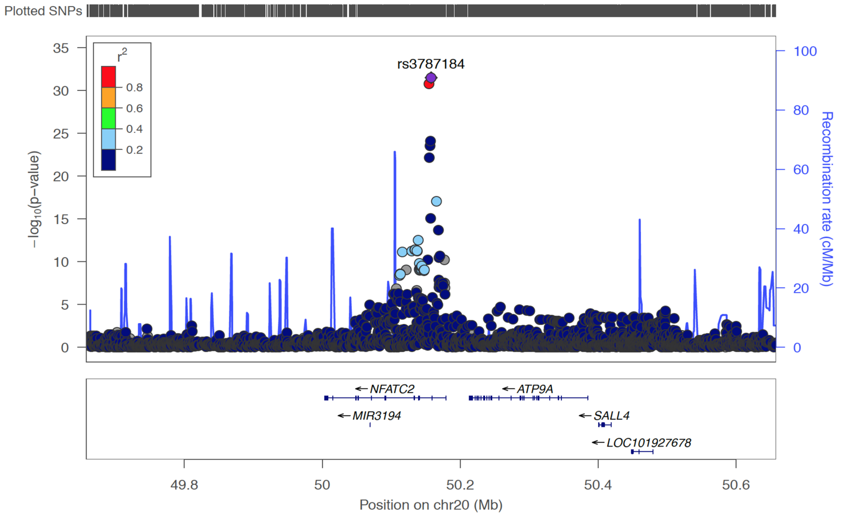

rs76602912

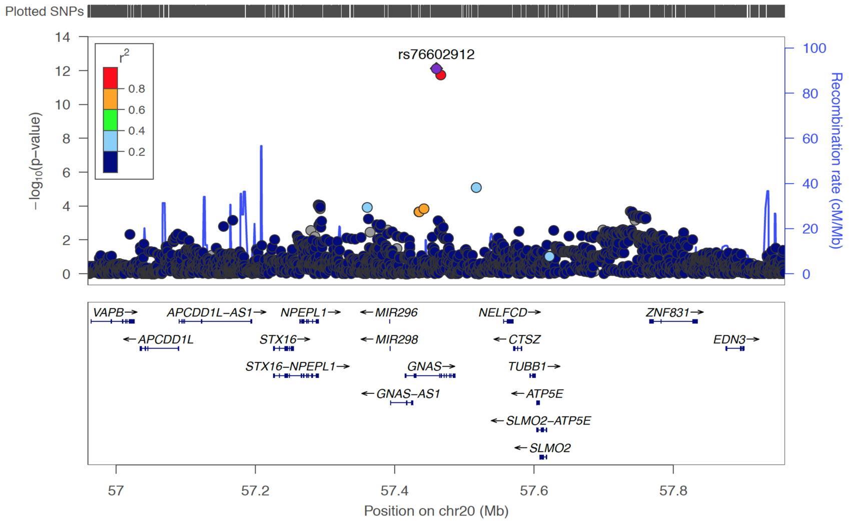

rs6062619

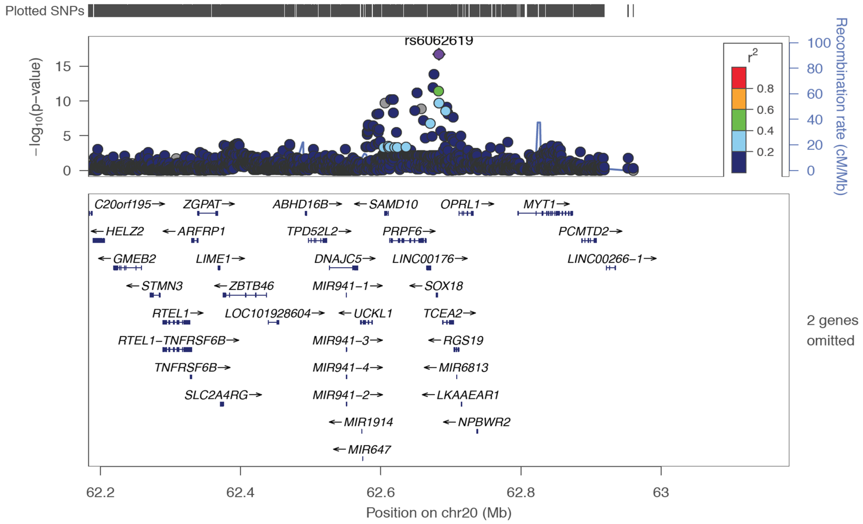

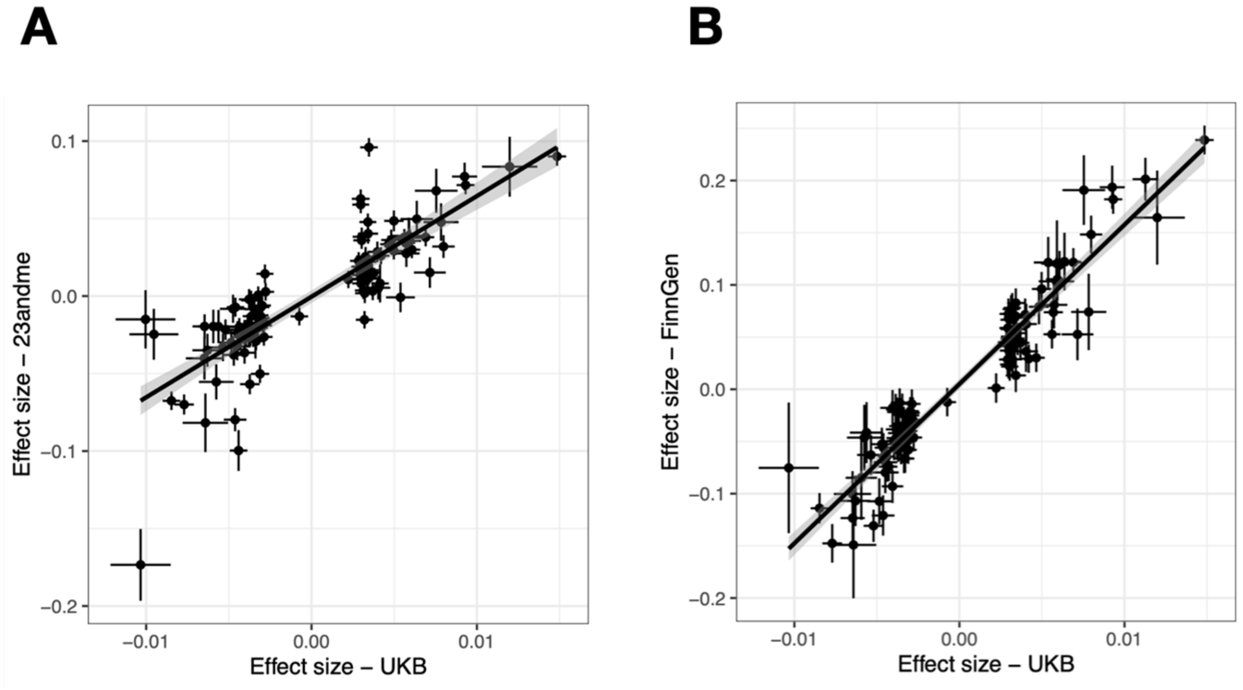

Supplementary Figure 3. High concordance between UKB, 23andMe, and FinnGen summary statistics. Effect size of each of 106 genome-wide significant index SNPs in the UK Biobank discovery cohort that were present in the 23andMe replication cohort are plotted against the effect sizes in 23andMe (panel A) and FinnGen (panel B). Error bars around the effect sizes (dots) signify standard error. The line of best fit is shown, with 95% confidence intervals shaded in grey.

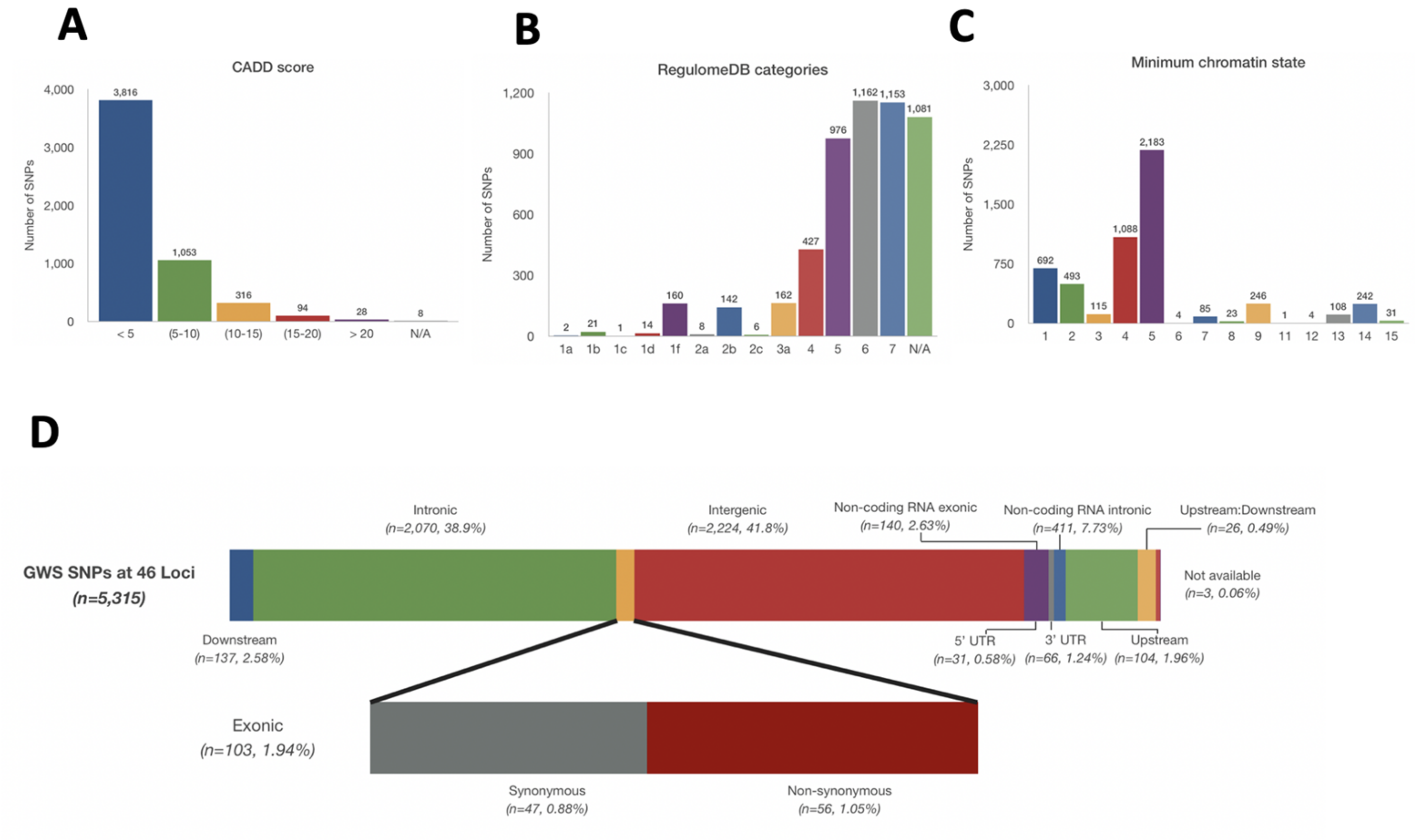

Supplementary Figure 4. Functional annotation of the 5,315 genome-wide significant SNPs at our 46 replicated loci. Functional consequences of the SNPs on genes were obtained by performing ANNOVAR gene-based annotation using Ensembl genes (build 85) in FUMA. A) CADD scores, B) RegulomeDB scores and C) 15-core chromatin state were annotated to all 5,315 SNPs in 1000G phase 3 by FUMA through matching chromosome, position, reference, and alternative alleles. D) Positional classification of the 5,315 SNPs.

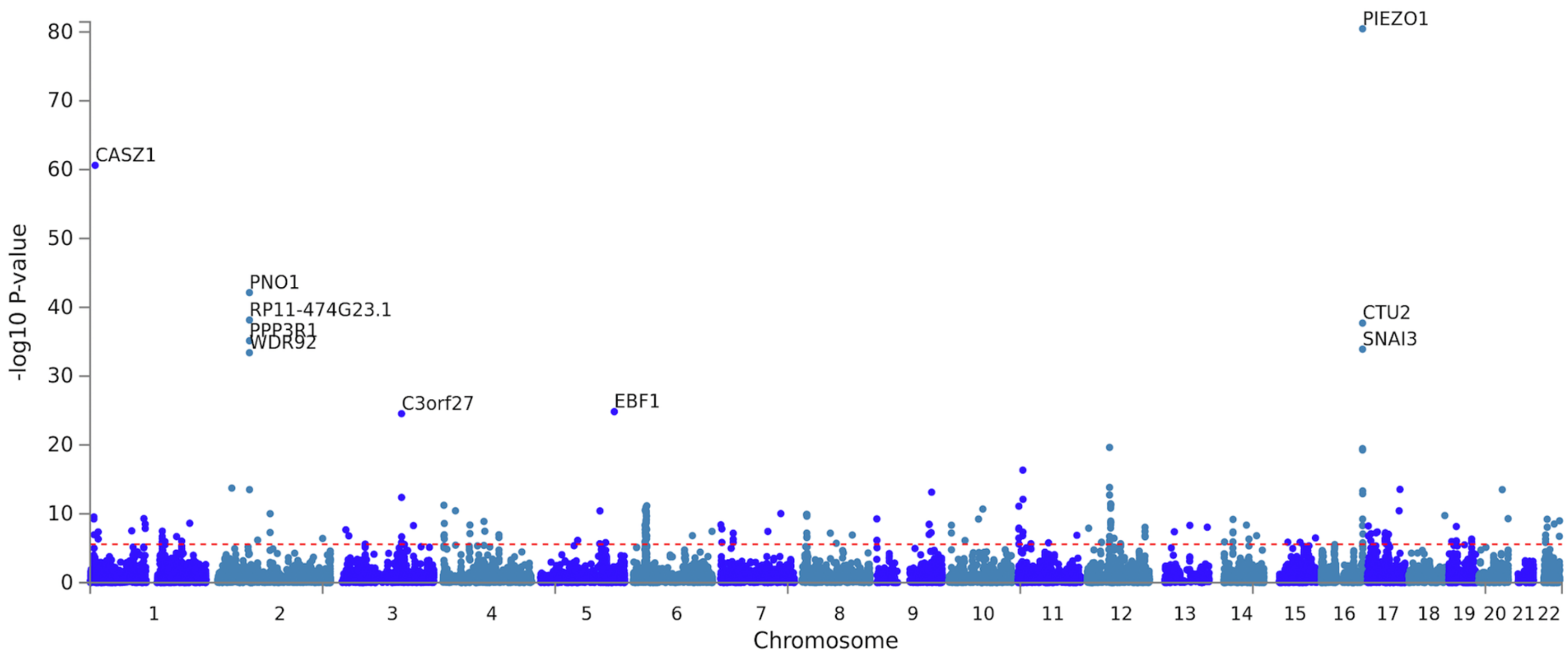

Supplementary Figure 5. MAGMA Gene-based association analysis Manhattan plot. Association results for varicose veins in MAGMA gene-based association analysis. The dotted red line indicates the threshold for genome-wide significance ($P < 2.68\times10^{-6}$) corrected for multiple testing. 248 genes reached genome-wide significance in this analysis, with the top-ten genes highlighted in this figure.

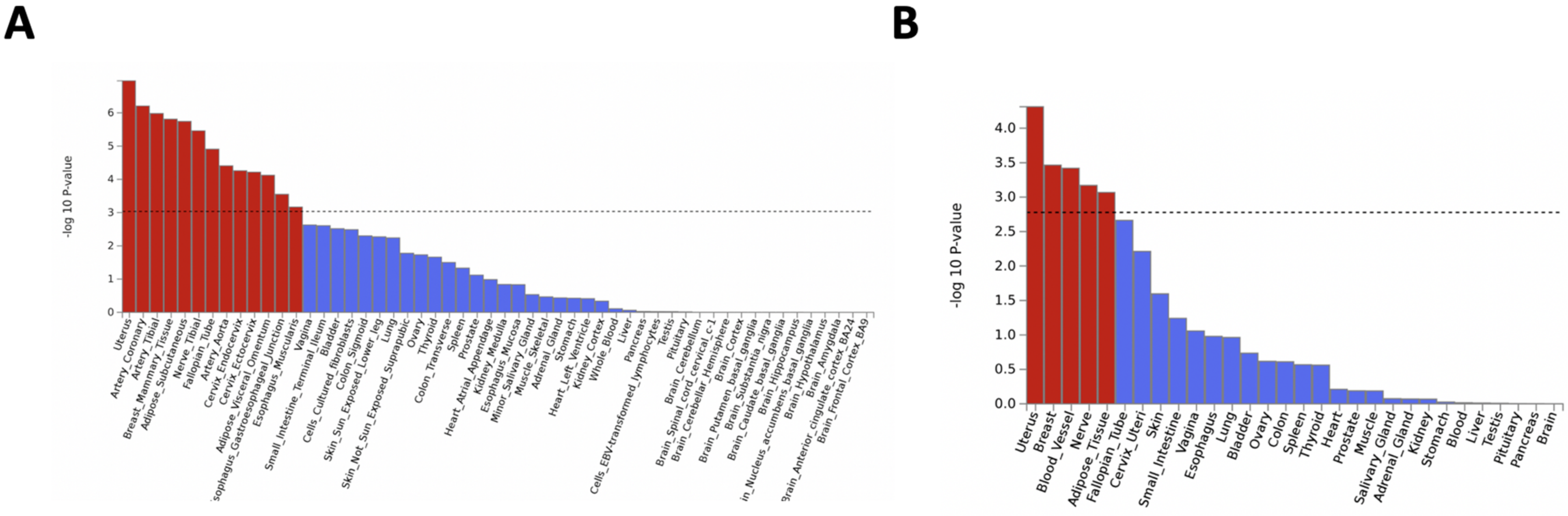

Supplementary Figure 6. MAGMA tissue expression analysis. MAGMA Tissue Expression Analysis of varicose veins GWAS-summary data, implemented in FUMA in A) 54 specific and B) 30 general tissue types. This analysis tests the relationship between highly-expressed genes in a specific tissue and the genetic associations from the GWAS. Gene-property analysis is performed using average expression of genes per tissue type as a gene covariate. Gene expression values are log2 transformed average RPKM (Read Per Kilobase Per Million) per tissue type after winsorization at 50, and are based on GTEx v8 RNA-Seq data across 54 specific tissue types and 30 general tissue types. The dotted line indicates the Bonferroni-corrected α level, and the tissues that meet this significance threshold are highlighted in red.

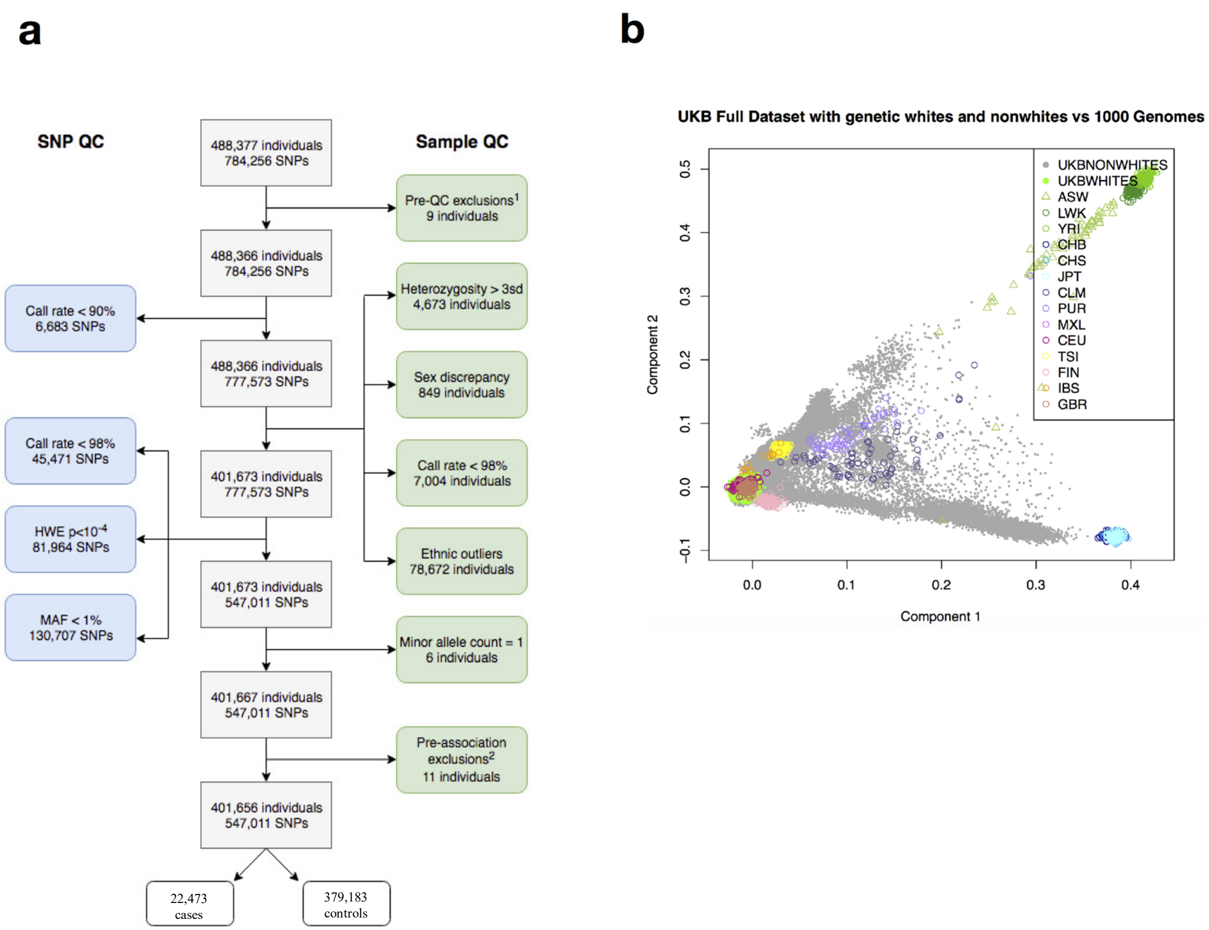

Supplementary Figure 7. Overview of Quality Control (QC). a, Flowchart summarising QC protocol. Excluded SNPs are in blue panels on the left and excluded individuals are in green panels on the right. [1]Pre-QC exclusions: 3 individuals with invalid IDs and sex, and 8 individuals who have withdrawn from UK Biobank were excluded prior to QC. [2]Pre-association exclusions: 11 individuals who were not present in UK Biobank's sample file accompanying the BGEN files were excluded prior to association. **b**, Principal Component Analysis (PCA) for demonstration of ethnicity of UK Biobank individuals. The UK Biobank cohort was merged with publicly available data from the 1000 Genomes Project and PCA was performed using flashpca. Individuals identified by UK Biobank as having white British ancestry are coloured in lime green, and the remaining UK Biobank individuals are in grey. In this graph of principal component 1 vs principal component 2, a near-perfect overlap can be seen between

the UK Biobank “white British” individuals and both GBR (British in England and Scotland - light brown) and CEU (Utah residents with Northern and Western European ancestry - magenta) individuals from the 1000 Genomes Project.
