# Supplementary material for: Genome-wide association analysis and replication in 810,625 individuals with varicose veins
Source: Nat Commun. 2022 Jun 2;13:3065. doi: 10.1038/s41467-022-30765-y (PMC9163161; doi:10.1038/s41467-022-30765-y)
Supplement: Supplementary file 3 — Description of Additional Supplementary Files [file 41467_2022_30765_MOESM3_ESM.pdf]

Description of Additional Supplementary Files

File Name: Supplementary Data 1
Description: **Genome-wide significant variants at all significant discovery loci.** 12,391 genome-wide significant variants ($P < 5\times10^{-8}$) at all 108 genomic risk loci identified in the discovery GWAS. The column headings pertain to: the SNP ID (rsID), chromosome, base position within NCBI Genome Build 37 (hg19), the effect allele, the alternate (non-effect) allele, the effect allele frequency in the study population, the imputation quality score; where 1 = genotyped SNP, the SNP effect size (BETA), the standard error of the BETA, and the GWAS association P-value. All variants are presented in ascending order according to chromosome and base position.

File Name: Supplementary Data 2
Description: Forty-nine variants at 46 susceptibility loci significantly associated with varicose veins.

File Name: Supplementary Data 3
Description: **Additional variants associated with varicose veins in discovery cohort.** 67 independent variants at 67 loci were genome-wide significant ($P < 5\times10^{-8}$) in the UK Biobank cohort, however, they either did not meet the Bonferroni-corrected threshold of $P < 4.72\times10^{-4}$ in the 23andMe replication cohort, or replication data were not available (for ten tested variants).

File Name: Supplementary Data 4
Description: **Genome-wide significant variants at the replicated susceptibility loci.** 5,315 genome-wide significant variants ($P < 5\times10^{-8}$) identified by FUMA SNP2GENE at 45 of 46 replicated genomic risk associated with varicose veins. The column headings pertain to: the unique ID of the variant, the SNP ID (rsID), chromosome, base position within NCBI Genome Build 37 (hg19), the effect allele, the alternate (non-effect) allele, the effect allele frequency in the study population, the SNP effect size (BETA), the standard error of the BETA, the nearest gene according to positional mapping in FUMA, the functionality of the SNP as derived from ANNOVAR, the Combined Annotation Dependent Depletion (CADD) score of the variant, the RegulomeDB (RDB) score of the variant, the minimum chromatin state of the variant.

File Name: Supplementary Data 5
Description: **Varicose veins-associated exonic variants at the replicated loci.** 103 genome-wide significant exonic SNPs at seventeen of the replicated varicose veins susceptibility loci were identified by FUMA SNP2GENE.

File Name: Supplementary Data 6
Description: **Predicted functional intronic and intergenic variants at the replicated loci.** 163 genome-wide significant intronic and intergenic variants predicted to be deleterious according to a CADD ≥ 12.37, as identified by FUMA SNP2GENE.

File Name: Supplementary Data 7
Description: **Genes mapped to the varicose veins associated loci using the four mapping strategies.** 237 unique genes were mapped to 39 of 46 replicated loci by one or more gene mapping strategies (see

Methods). 204 genes were mapped via positional mapping in FUMA, 80 genes were mapped via eQTL mapping in FUMA, 117 genes were mapped using MAGMA and 14 genes were mapped using summary-based Mendelian randomisation. In total, 61 unique genes were mapped to novel loci. Overlap between the four different mapping strategies is shown.

File Name: Supplementary Data 8
Description: **Genome-wide gene-based association analysis in MAGMA.** 248 protein-coding genes met the threshold for genome-wide significance ($p<2.67\times10^{-6}$, 0.05/18,733) in this analysis. 117 of the 248 genes lay within our replicated loci and are highlighted in red.

File Name: Supplementary Data 9
Description: **Summary-based Mendelian Randomisation (SMR) using eQTL data from GTEx v7 tibial artery.** The 25 probes (genes) that met the Bonferroni-corrected significance threshold $P_{SMR} < 1.01\times10^{-5}$ (0.05/4,946) and passed the HEIDI test ($P_{HEIDI} \geq 1.12\times10^{-3}$) (0.05/44)) are shown. Twelve probes mapped to our replicated loci.

File Name: Supplementary Data 10
Description:**. Phenome-wide associations of 49 replicated VVs-associated variants in Open Target Genetics.** A PheWAS search was performed for the 49 replicated VVs-associated variants in the Open Targets Genetics platform (accessed 25 Feb 2022). The variant rsIDs are highlighted in red. All traits with P < 0.005 are shown.

File Name: Supplementary Data 11
Description: **Enriched drug pathways from the drug target enrichment analysis.** Mapped genes were interrogated with the Open Targets Platform to enrich drug pathways relating to the identified gene targets. The 200 gene targets identified by the Open Targets Platform mapped to 622 drug pathways, of which, 42 reached a nominal significance P < 0.05 and are shown below (no adjustments were made for multiple testing).

File Name: Supplementary Data 12
Description: **Tractability information for targets in the drug-target enrichment analysis.** The 237 mapped genes were interrogated within the Open Targets Platform to determine their tractability to small molecule and antibody targeting. 200 genes targets were identified by the Open Targets Platform of which, tractability information was available for 105 gene targets (left column).

File Name: Supplementary Data 13
Description:**. Pharmacologically active targets identified in drug-target enrichment.** Of the 200 varicose veins-associated genes targets identified by the Open Targets Platform, eight gene targets have known pharmaceutical interactions and are presently, or in the past have been, investigated in clinical trials in different phases for the treatment of several diseases. Diseases shown below are those relating to vascular disorders.

File Name: Supplementary Data 14

Description: **Functional categories of the gene clusters.** The varicose veins susceptibility loci map to genes implicated in five functional categories. Several genes map to more than one category. Genes not associated with varicose veins previously are highlighted in bold.

File Name: Supplementary Data 15
Description: **MAGMA gene set analysis.** Gene sets were obtained from MsigDB v7.0 and total of 15,496 gene sets (Curated gene sets: 5,500, GO terms: 9,996) were tested. Curated gene sets consists of 9 data resources including KEGG, Reactome and BioCarta (http://software.broadinstitute.org/gsea/msigdb/collection_details.jsp#C2 for details). GO terms consists of three categories, biological processes (bp), cellular components (cc) and molecular functions (mf). All parameters were set as default (competitive test). Gene sets are arranged in order of ascending P-value, with a P-value $< 3.23\times10^{-6}$ indicative of a significant gene set, accounting for multiple testing (0.05/15,496).
