# Supplementary material for: Genome-wide association analysis and replication in 810,625 individuals with varicose veins
Source: Nat Commun. 2022 Jun 2;13:3065. doi: 10.1038/s41467-022-30765-y (PMC9163161; doi:10.1038/s41467-022-30765-y)
Supplement: Supplementary file 19 — Reporting Summary [file 41467_2022_30765_MOESM19_ESM.pdf]

nature portfolio

Corresponding author(s): Dominic Furniss

Last updated by author(s): Apr 7, 2022

Reporting Summary

Nature Portfolio wishes to improve the reproducibility of the work that we publish. This form provides structure for consistency and transparency in reporting. For further information on Nature Portfolio policies, see our Editorial Policies and the Editorial Policy Checklist.

Statistics

For all statistical analyses, confirm that the following items are present in the figure legend, table legend, main text, or Methods section.

n/a	Confirmed	
☐	☒	The exact sample size (n) for each experimental group/condition, given as a discrete number and unit of measurement
☒	☐	A statement on whether measurements were taken from distinct samples or whether the same sample was measured repeatedly
☐	☒	The statistical test(s) used AND whether they are one- or two-sided<br>*Only common tests should be described solely by name; describe more complex techniques in the Methods section.*
☐	☒	A description of all covariates tested
☐	☒	A description of any assumptions or corrections, such as tests of normality and adjustment for multiple comparisons
☐	☒	A full description of the statistical parameters including central tendency (e.g. means) or other basic estimates (e.g. regression coefficient) AND variation (e.g. standard deviation) or associated estimates of uncertainty (e.g. confidence intervals)
☐	☒	For null hypothesis testing, the test statistic (e.g. F, t, r) with confidence intervals, effect sizes, degrees of freedom and P value noted<br>*Give P values as exact values whenever suitable.*
☒	☐	For Bayesian analysis, information on the choice of priors and Markov chain Monte Carlo settings
☒	☐	For hierarchical and complex designs, identification of the appropriate level for tests and full reporting of outcomes
☐	☒	Estimates of effect sizes (e.g. Cohen's d, Pearson's r), indicating how they were calculated

Our web collection on statistics for biologists contains articles on many of the points above.

Software and code

Policy information about availability of computer code

Data collection	No software was used for data collection.

Data analysis

We used the following publicly available software for data analysis:

PLINK v1.9 for quality control of UK Biobank data
R v3.3.1
FlashPCA v2.0
BOLT-LMM v2.3
Eagle2 v2.3
GWAMA v2.2.2
FUMA v1.3.3
MAGMA v1.07
Polyfun-SuSiE v0.11.92
echolocatoR v0.2.2
SMR v0.710
XGR v1.1.3
BOLT-REML v2.3
LDSC v1.0.1
Hail v0.2.77
gnomAD package v0.4.0
Bigsnpr package v1.9.11
Plink v2.00 alpha for polygenic risk score analyses
TwoSampleMR v.0.5.6

For manuscripts utilizing custom algorithms or software that are central to the research but not yet described in published literature, software must be made available to editors and reviewers. We strongly encourage code deposition in a community repository (e.g. GitHub). See the Nature Portfolio guidelines for submitting code & software for further information.

Data

Policy information about availability of data

All manuscripts must include a data availability statement. This statement should provide the following information, where applicable:

- Accession codes, unique identifiers, or web links for publicly available datasets
- A description of any restrictions on data availability
- For clinical datasets or third party data, please ensure that the statement adheres to our policy

Discovery GWAS summary statistics from UK Biobank have been deposited in the Oxford University Research Archive and are available for download at: https://doi.org/10.5287/bodleian:8J26woZQg. Full UK Biobank data can be accessed by direct application to UK Biobank. Genotype data for 23andMe research participants have not been deposited in public repositories, as consent for this was not obtained in the study protocol. Summary statistics can be accessed from 23andMe by qualified researchers who enter into agreement with 23andMe that protects subjects' confidentiality. Investigators wishing to collaborate with 23andMe can email dataset-request@23andme.com or apply via the 23andMe research website (www.23andme.com/en-gb/research).

The following publicly available data sources were used in this study:

1000 Genomes Project (www.1000genomes.org)
HRC www.haplotype-reference-consortium.org
UK10000/1000 Genomes Phase 3 Panel https://www.uk10k.org/data_access.html
gnomAD https://gnomad.broadinstitute.org/
Ensembl genome browser www.ensembl.org/index.html
Regulome DB: https://regulomedb.org/regulome-search/
ANNOVAR: (https://annovar.openbioinformatics.org/en/latest/
GTEx portal: www.gtexportal.org/home/
MSigDB: http://www.gsea-msigdb.org/gsea/msigdb/collections.jsp
LD Hub: https://ldsc.broadinstitute.org/
Open Targets Genetics: https://genetics.opentargets.org/
FinnGen: https://www.finngen.fi/en
FinnGen varicose vein phenotype definition: https://risteys.finngen.fi/phenocode/I9_VARICVE
OpenGWAS project: https://gwas.mrcieu.ac.uk/
Open Targets platform: https://platform.opentargets.org/

Field-specific reporting

Please select the one below that is the best fit for your research. If you are not sure, read the appropriate sections before making your selection.

☒ Life sciences ☐ Behavioural & social sciences ☐ Ecological, evolutionary & environmental sciences

For a reference copy of the document with all sections, see nature.com/documents/nr-reporting-summary-flat.pdf

Life sciences study design

All studies must disclose on these points even when the disclosure is negative.

Sample size	No sample size calculations were performed. To maximise power, we used all available varicose veins cases within the post-QC UK Biobank dataset, and assigned the remaining participants as controls.
Data exclusions	Our QC methodology detailing sample exclusions is described in the article. Exclusion criteria were based on: heterozygosity > 3 S.D. from the mean, sex discrepancy between genotypic and self-declared sex, SNP call rate <98%, ethnicity (analysis was limited to individuals of white British ancestry), and visual outliers when autosomal heterozygosity was platted against call rate. These exclusion criteria were pre-established.
Replication	Replication was successfully performed in an independent dataset.
Randomization	This is not a clinical trial - randomisation is not relevant.
Blinding	This is not a clinical trial - blinding is not relevant.

Reporting for specific materials, systems and methods

We require information from authors about some types of materials, experimental systems and methods used in many studies. Here, indicate whether each material, system or method listed is relevant to your study. If you are not sure if a list item applies to your research, read the appropriate section before selecting a response.

Materials & experimental systems

n/a	Involved in the study	
☒	☐	Antibodies
☒	☐	Eukaryotic cell lines
☒	☐	Palaeontology and archaeology
☒	☐	Animals and other organisms
☐	☒	Human research participants
☒	☐	Clinical data
☒	☐	Dual use research of concern

Methods

n/a	Involved in the study	
☒	☐	ChIP-seq
☒	☐	Flow cytometry
☒	☐	MRI-based neuroimaging

Human research participants

Policy information about studies involving human research participants

Population characteristics	The UK Biobank is a population-level resource comprising a prospective cohort of approximately 500,000 participants, recruited at age 40-69 years, at 22 centres across England, Scotland, and Wales, between 2006 and 2010. Participants underwent whole-genome genotyping and data linkage with their medical records was performed to permit deep phenotyping. For the discovery GWAS, we identified 22,473 varicose veins cases and 379,183 controls.
Recruitment	UK Biobank participants were recruited at age 40-69 years, at 22 centres across England, Scotland, and Wales, between 2006 and 2010. Participants underwent whole-genome genotyping and data linkage with their medical records was performed to permit deep phenotyping. There is a likely to be a bias towards recruitment of healthier-than-average participants to a population-based cohort study of this kind, but this should not bias the findings from the GWAS, as both cases and controls were drawn from the same population.
Ethics oversight	UK Biobank obtained ethical approval from the North West Multi-Centre Research Ethics Committee (MREC) (11/NW/0382) to collect and disseminate data and samples from participants (for more details: ukbiobank.ac.uk/ethics). This study was conducted under UK Biobank study ID 22572. All participants provided informed consent for their genotype data to be used for this research. The consent procedures for UK Biobank are provided elsewhere (for more details: www.ukbiobank.ac.uk/).

Note that full information on the approval of the study protocol must also be provided in the manuscript.
